# Multiple genome alignment for identifying the core structure among moderately related microbial genomes

**DOI:** 10.1186/1471-2164-9-515

**Published:** 2008-10-31

**Authors:** Ikuo Uchiyama

**Affiliations:** 1 Department of Theoretical Biology, National Institute for Basic Biology, National Institutes of Natural Sciences, Nishigonaka 38, Myodaiji, Okazaki, Aichi 444-8585 Japan

## Abstract

**Background:**

Identifying the set of intrinsically conserved genes, or the genomic core, among related genomes is crucial for understanding prokaryotic genomes where horizontal gene transfers are common. Although core genome identification appears to be obvious among very closely related genomes, it becomes more difficult when more distantly related genomes are compared. Here, we consider the core structure as a set of sufficiently long segments in which gene orders are conserved so that they are likely to have been inherited mainly through vertical transfer, and developed a method for identifying the core structure by finding the order of pre-identified orthologous groups (OGs) that maximally retains the conserved gene orders.

**Results:**

The method was applied to genome comparisons of two well-characterized families, *Bacillaceae *and *Enterobacteriaceae*, and identified their core structures comprising 1438 and 2125 OGs, respectively. The core sets contained most of the essential genes and their related genes, which were primarily included in the intersection of the two core sets comprising around 700 OGs. The definition of the genomic core based on gene order conservation was demonstrated to be more robust than the simpler approach based only on gene conservation. We also investigated the core structures in terms of G+C content homogeneity and phylogenetic congruence, and found that the core genes primarily exhibited the expected characteristic, *i.e*., being indigenous and sharing the same history, more than the non-core genes.

**Conclusion:**

The results demonstrate that our strategy of genome alignment based on gene order conservation can provide an effective approach to identify the genomic core among moderately related microbial genomes.

## Background

A growing body of evidence is supporting the idea that horizontal gene transfers (HGT) have played a significant role in prokaryotic genome evolution [1-6]. Although these observations have stimulated researchers to develop a new paradigm of HGT-driven reticulate evolution that challenges the traditional tree-based phylogeny concept [7-9], it can be argued that prokaryotic phylogeny can still be inferred using a certain subset of genes ("core genes") that have mainly transferred vertically throughout evolution [10-12]. In fact, the genes constituting a prokaryotic genome appear to be divided into two classes: a "core gene pool" that comprises intrinsic genes encoding the proteins of basic cellular functions, and a "flexible gene pool" that comprises HGT-acquired genes encoding proteins which function under particular conditions, such as genomic islands [13]. Therefore, the identification of the genomic core conserved among each taxonomic group is crucial, not only for establishing the identity of each taxonomic group, but also for understanding prokaryotic diversity and evolution. Moreover, in a practical sense, the genomic core concept should also play a key role in summarizing genomic databases, which continue to grow explosively [14,15].

However, the best way to define core genomes is yet to be established. Although the term "core genome" has been used in various contexts, in the context of intraspecific comparisons, "core genome" is typically defined as a set of genes shared by all strains, while "pan-genome" is defined as the union of genes contained in all the strains considered [16-18]. This definition of "core genome" can also be applied to genus-level comparisons [19], and actually, similar types of analyses have been conducted for comparisons of even more distantly related genomes [20,21]. However, with such strict criteria, the number of core genes often decreases excessively as the number of target genomes increases [22], since the sets of genes required for life can vary between organisms living in different environments. The problem can be avoided by using a relaxed conservation criterion rather than strict universality [22], but the problem of how to define a biologically meaningful core still remains. Moreover, despite several studies on how to establish plausible orthologous groups [23-26], the problem of identifying genuine orthologs is still difficult, especially for the comparison of prokaryotic genomes where horizontal transfers are common. In fact, in a strict sense, "genuine ortholog" is only meaningful when the genes have been transmitted vertically, and in that sense, "core genome" and "genuine ortholog" are closely related concepts.

Due to the accumulation of microbial genomic data, we can now compare genomes at various levels of relatedness [14]. In this work, we focused on comparisons of moderately related genomes (more specifically, in the taxonomic rank *family*), which we consider to be key for understanding prokaryotic evolution since they bridge the gap between the knowledge of short-term evolution and that of long-term evolution. Among such genomes, typically the gene orders are partially conserved, and this information can be used to identify genuine orthologs (except in the case where HGT is included by homologous recombination or independent insertions of segments carrying the same set of genes at orthologous loci in different genomes; see the Discussion section).

**Figure 1:**
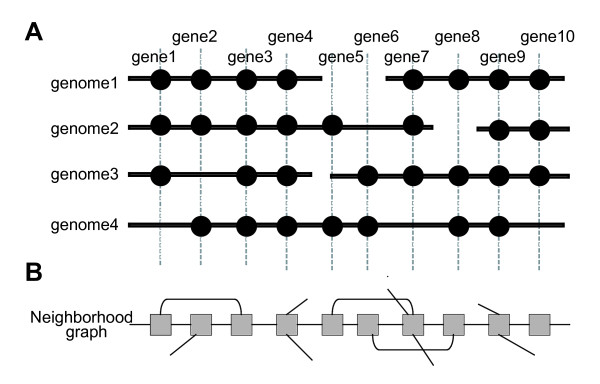
**Schematic illustration of the core genome construction procedure.** (A) A "core genome alignment" is defined as the order of pre-identified conserved OGs (vertical lines) which retains to the greatest possible extent the conserved neighborhood relationships on the chromosomes (horizontal lines). (B) A neighborhood graph of (A), where a node (square) represents an OG, and an edge (line) represents a neighborhood relationship between OGs. Here, for simplicity, only OGs that are directly adjacent to each other are connected, but, actually, the CoreAligner procedure connects all pairs of OGs that are within *MAX_GAP *genes of each other in at least a given ratio (*NBR_CONS_RATIO*) of the total number of genomes.

Here, we consider the structural core gene set, or simply the core structure, of moderately related genomes; this core gene set is defined as the set of sufficiently long consecutive genomic segments in which gene orders are conserved among multiple genomes so that they are likely to have been inherited from a common ancestor mainly through vertical transfer (hereafter, unless otherwise stated, by "core" we mean this type of core gene set, whereas we use "universal" to refer to the genes shared by all the genomes considered). For this purpose, we developed a graph-based algorithm for aligning conserved regions of multiple genomes, which finds the order of pre-identified gene families that retains to the greatest possible extent the conserved gene orders (Figure 1). To confirm that our definition of the core structure is indeed biologically meaningful, we applied our method to genome comparisons of two well-characterized families, *Bacillaceae *and *Enterobacteriaceae*, and characterized the resulting core structures in terms of gene functions, essentiality, G+C content homogeneity and phylogenetic congruence.

## Results

### CoreAligner: procedure for constructing a core genome alignment

Our program for constructing core structures, named CoreAligner, requires a set of well-conserved orthologous groups (OGs). Here, we compiled a set of OGs using the DomClust algorithm [24] on the MBGD server [14], and considered an OG as "conserved" when it was present in at least half of the genomes (the parameter *CONS_RATIO *= 0.5). Next, conserved neighborhood pairs were extracted from the pairs of conserved OGs. We considered that two OGs are in a neighborhood in a given genome if they are located within 20 genes (*MAX_GAP *= 20) in that genome, and that the OG pair is conserved if the two OGs in that pair are in the same neighborhood in at least half of the genomes (the parameter *NBR_CONS_RATIO*, which was set equal to *CONS_RATIO *throughout this work). A neighborhood graph, *G*_0 _= (*V*, *E*), was then constructed with the set of conserved OGs, *V*, and the set of conserved neighborhood relationships, *E*.

Our algorithm for constructing the alignments of the core genome structures is based on finding the longest path of the conserved neighborhood graph (Figure 1). A similar algorithm has been previously developed [27] mainly for identifying much shorter but more widely conserved gene clusters such as operons and über-operons [28], but unlike their method, our method considers not only genes in the same direction but also those in the opposite direction as neighboring genes, and thereby generally generates longer alignments. In addition, our method uses the dynamic programming (DP) algorithm for calculating the longest path. To apply the DP algorithm, we devised a heuristic scheme comprising a series of preprocessing procedures to convert the initial conserved neighborhood graph, *G*_0_, into a directed acyclic graph, *G*_3 _(see Methods). After that, the extracted longest path is added to the core structure when the path consists of more than 10 OGs (*MIN_CLUSTER *= 10) and at least half of the genes (OGs) in that path are present in every genome (*SP_COVER *= 0.5). The procedure is repeated to find the next longest path in the remaining graph and the iteration is continued until all such paths are found.

### Core genome structure of *Bacillaceae *and *Enterobacteriaceae*

We used two sets of moderately related genomes: eight species belonging to the family *Bacillaceae *and eight species belonging to the family *Enterobacteriaceae *(Table 1), among which gene orders are partially conserved (see Additional file 1 for pairwise dotplots). In addition, we used *Staphylococcus aureus *and *Vibrio cholerae *as outgroup species. We excluded endosymbionts with extremely reduced genomes such as *Buchnera *and *Wigglesworthia *from the *Enterobacteriaceae *set, although we included in the analysis an endosymbiont, *Sodalis glossinidius*, that has a chromosome of more than 4 Mb containing 2432 open reading frames (ORFs) [29]. On the basis of the pairwise comparisons among these genomes (Additional file 1), we considered that two pairs of genomes (*Bacillus subtilis *and *Bacillus licheniformis*; *Bacillus anthracis *and *Bacillus cereus*) in the *Bacillaceae *set and a set of three genomes (*Escherichia coli*, *Salmonella enterica *and *Enterobacter *sp. 638) in the *Enterobacteriaceae *set are related sufficiently closely that they can be counted only once (Table 1). Consequently, the effective number of genomes was six in both sets.

**Table 1:** Genomic data used in this work.

***Bacillaceae***	Group	Abbrev.	Accession No.	CDS	GC%
*Bacillus subtilis *168	1*	*B. sub *(bsu)	NC_000964.2	4105	43.5
*Bacillus licheniformis *ATCC 14580	1	*B. lic *(bli)	NC_006270.2	4152	46.2
*Bacillus halodurans *C-125		*B. hal *(bha)	NC_002570.2	4066	43.7
*Bacillus clausii *KSM-K16		*B. cla *(bcl)	NC_006582.1	4096	44.8
*Bacillus anthracis *Ames	2*	*B. ant *(ban)	NC_003997.3	5311	35.4
*Bacillus cereus *14579	2	*B. cer *(bce)	NC_004722.1	5234	35.3
*Geobacillus kaustophilus *HTA426		*G. kau *(gka)	NC_006510.1	3498	52.0
*Oceanobacillus iheyensis *HTE831		*O. ihe *(oih)	NC_004193.1	3500	35.7
(outgroup)
*Staphylococcus aureus *N315		*S. aur *(sau)	NC_002745.2	2588	32.8

***Enterobacteriaceae***					

*Escherichia coli *K-12	3*	*E. col *(eco)	NC_000913.2	4131	50.8
*Salmonella enterica *CT18	3	*S. ent *(sty)	NC_003198.1	4395	51.9
*Enterobacter *sp. 638	3	*Enter *(ent)	NC_009436.1	4115	52.9
*Erwinia carotovora *SCR11043		*E. car *(eca)	NC_004547.2	4472	51.0
*Photorhabdus luminescens *TTO1		*P. lum *(plu)	NC_005126.1	4683	42.8
*Sodalis glossinidius *2516		*S. glo *(sgl)	NC_007712.1	2432	54.5
*Serratia proteamaculans 568*		*S. pro *(spe)	NC_009832.1	4891	55.0
*Yersinia pestis *CO92		*Y. pes *(ype)	NC_003143.1	3885	47.6
(outgroup)
*Vibrio cholerae *N16961		*V. cho *(vch)	NC_002506.1 NC_002505.1	3835	47.5

In the Group column, organisms with the same number are those considered to be so close that they are counted only once; organisms with an asterisk are representative genomes for each of the closely related genome groups, which were used in the robustness test and the phylogenetic congruence test. In the Abbrev column, the abbreviated names used in this paper are shown in italic; the names in parentheses are the abbreviated names used in KEGG and MBGD.

**Figure 2:**
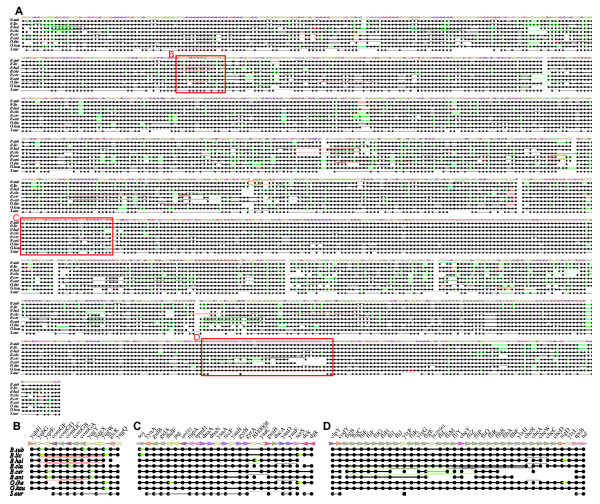
**Core genome alignment of the *Bacillaceae *dataset.** (A) Entire alignment; (B) a region around the *comG *operon that contains a local rearrangement; (C) a region around the replication origin; (D) a cluster of motility-related genes in which some of the genes are missing in the *B. anthracis *and *B. cereus *genomes. The rows and columns represent genomes and OGs, respectively. A black line represents a direct adjacency, a green line represents a non-adjacent neighborhood (*i.e*., there are insertions), and a red line represents an inversion. See Additional file 2 for the complete figures for both *Bacillaceae *and *Enterobacteriaceae*, which also contain the name of each OG.

By applying the CoreAligner program to these datasets, we obtained the alignments of the core structures of the *Bacillaceae *and *Enterobacteriaceae *genomes comprising 1438 and 2125 OGs, respectively (Figure 2 and Additional file 2; for a full list of core genes, see Additional file 3). The number of *Bacillaceae *core genes is about a third of the number of genes in the *B. subtilis *genome (4105), and the number of *Enterobacteriaceae *core genes is about half of the number of genes in the *E. coli *genome (4237), although the actual numbers of core genes and total genes are different among the organisms.

Table 2 shows the number of deleted core OGs for each organism. The number of core genes is generally not correlated with the total number of genes in that genome. In fact, although the *B. anthracis *genome (5311) contains many more genes than the *Geobacillus kaustophilus *genome (3498), *B. anthracis *has lost many more core genes than *G. kaustophilus*; this is partly due to the partial loss of the motility-related genes in the former genome (see Figure 2D). This indicates that the substantial size difference among genomes is primarily accounted for by the non-core genes, and any event yielding such a size difference affects the core genome structure to a lesser extent. On the other hand, among the *Enterobacteriaceae*, the *S. glossinidius *genome, which has the smallest number of genes, has lost the largest number of core genes; the number of its core genes is even smaller than that of the outgroup species *V. cholerae*. The genome sequence of *S. glossinidius *exhibits massive genome erosion, which supports the idea of the recent establishment of its endosymbiosis with the tsetse fly [29]. In this case, this erosion process appears to have had an influence even upon the reduction of the core structure. Similarly, the *Serratia proteamaculans *genome, which has the largest number of genes, also has the largest number of core genes among the *Enterobacteriaceae *(Table 2), although it is unclear whether or not this extremely high conservation of the core structure in the *S. proteamaculans *genome is indeed related to its large genome size.

**Table 2:** The number of deleted core orthologous groups in each genome.

*Bacillaceae *(1438 core OGs)		*Enterobacteriaceae *(2125 core OGs)	
*B. subtilis*	76	*E. coli*	125
*B. licheniformis*	51	*S. enterica*	124
*B. halodurans*	80	*Enterobacter *sp.	117
*B. clausii*	142	*E. carotovora*	119
*B. anthracis*	138	*P. luminescens*	362
*B. cereus*	141	*S. glossinidius*	666
*G. kaustophilus*	82	*S. proteamaculans*	26
*O. iheyensis*	173	*Y. pestis*	158

*S. aureus*	555	*V. cholerae*	539

**Figure 3:**
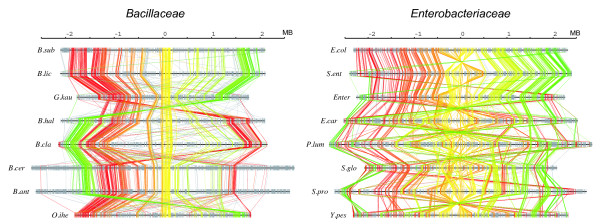
**Chromosomal arrangements of the core genes along each genome in *Bacillaceae *(left) and *Enterobacteriaceae *(right).** Here, only OGs that are universally conserved in a one-to-one correspondence are connected. Core OGs are colored from red to yellow to green according to the chromosomal positions in the reference genomes (*B. subtilis *and *E. coli*), and non-core OGs are drawn in grey. Unique genes in each genome are drawn as short vertical bars. Each chromosome is arranged so that the replication origin is located at the center.

The chromosomal arrangements of the core structures are somewhat different between these datasets (Figure 3). Especially, a remarkable conservation was observed in the chromosomal arrangement of the *Bacillaceae *core structure: the core genes are highly clustered and their overall arrangements are well conserved, except some symmetrical inversions near the replication terminus, a typical pattern of bacterial chromosomal rearrangement [30]. In contrast, the sizes of the gaps between the core regions vary substantially among genomes, and most of the species-specific genes are inserted in these flexible regions. On the other hand, the core genes of *Enterobacteriaceae *are less clustered and more highly shuffled, although from the sequence comparisons they generally appear to be more closely related to each other than the *Bacillaceae *genomes (see Additional file 1, upper right). Nonetheless, they also primarily show the typical symmetrical inversion pattern around the replication origin.

### Functional characteristics of the structural core gene sets

Next, we examined what kinds of genes are included in the core gene sets. We considered the *B. subtilis *and *E. coli *genomes as reference genomes and characterized each gene in these genomes using the functional categories defined in the KEGG Orthology database [31]. We also categorized each gene in these genomes into the following six classes: A) universal core, B) conserved core C) universal non-core, D) conserved non-core, E) non-conserved and non-unique, and F) unique, where "universal" means that the genes are conserved in all the genomes tested, "conserved" means that the genes are conserved in at least half of the genomes examined (the condition included in the definition of "core"), and "unique" means that the genes are unique to the reference genome (*B. subtilis *or *E. coli*); thus, "universal non-core" genes, for example, are genes conserved in all genomes but not included in the core set (i.e., syntenically not conserved).

**Figure 4:**
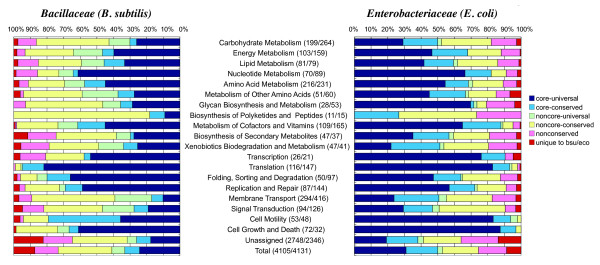
**Proportion of core and other classes in each KEGG functional category in the reference genome.** The numbers in parentheses after each category name are the numbers of genes in *B. subtilis *and *E. coli*, respectively.

Figure 4 shows the proportion of these six classes for each KEGG functional category. Although the overall proportions of core genes (A+B) are quite different between these families, the two graphs primarily exhibit a similar tendency: the functional categories related to primary metabolism, genetic information processing and cellular processes generally contain a higher proportion of core genes, while the categories of membrane transport, signal transduction and secondary metabolism contain a lower proportion thereof. Spearman's rank correlation coefficient between the two 20-dimensional vectors of the core gene proportions is *ρ *= 0.86, which confirms the high similarity of these distributions. Since those categories with a smaller core proportion mentioned above are likely to be related to adaptation to specific environments, this observation supports the notion that the genes included in the core structures indeed tend to play core functional roles.

To see to what extent the core gene sets cover the functionally important genes, we examined the existence of the essential gene sets that were identified from the systematic gene deletion experiments in *B. subtilis *[32] and *E. coli *[33]. In total, 251 out of 271 essential genes (92.6%) were included in the *B. subtilis *core gene set, and 275 out of 297 essential genes (92.6%) were included in the *E. coli *core gene set (Additional file 4). Therefore, most of the essential genes were included in our core gene sets in both cases. In effect, the coverage of the essential genes in the core gene sets might be even larger, because most of the non-core essential genes are not universally conserved. For example, among the *B. subtilis *essential genes that are not included in the core gene set are the cell-wall teichoic-acid biosynthesis genes (*tag*), the lack of whose orthologs in *G. kaustophilus *genome has been previously reported [34], suggesting that the essentiality of this system is not universal. A similar conclusion can be drawn from the comparison of the two independently identified *E. coli *essential gene sets [33,35]. When we consider the intersection of the essential gene set of Baba and that of Kato, the coverage increases up to 98.1% (256 out of 261 essential genes; genes without an asterisk in Additional file 4).

**Figure 5:**
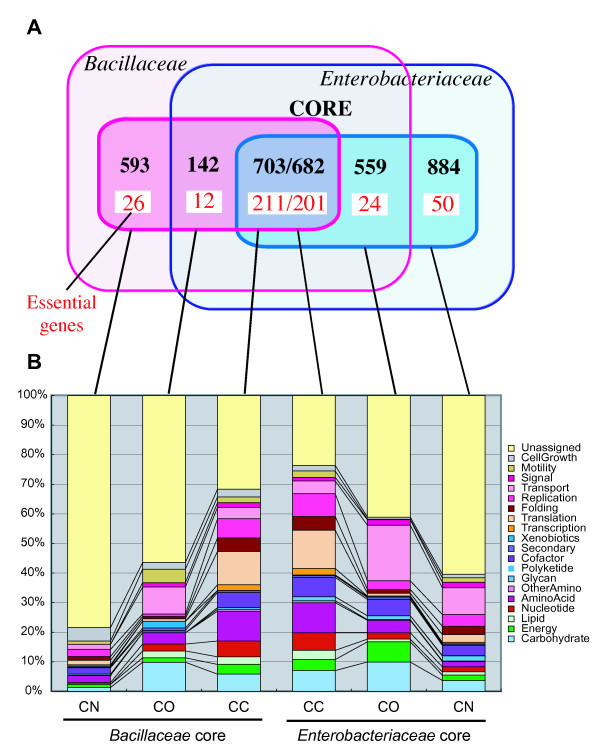
**Overlap between the core OG sets of *Bacillaceae *and *Enterobacteriaceae*.** (A) A Venn diagram showing the number of core OGs defined in *Bacillaceae *and *Enterobacteriaceae *that overlap each other. For each family, the outer circle indicates the entire gene set, and the inner circle indicates the core gene set. The red numbers indicate the number of essential genes in *B. subtilis *and *E. coli*. (B) Functional breakdown of each subtype defined in (A): CC core to core; CO, core to non-core ortholog; CN, core to none. The legend shows the abbreviated names of the KEGG functional categories. For the full category names, see Figure 4.

Actually, the core gene sets of *Bacillaceae *and *Enterobacteriaceae *share some common orthologs. To visualize this, we identified the orthologous relationships between these datasets by applying the DomClust program to the combined dataset (Figure 5A). The two core gene sets share 637 combined OGs that contain 703 and 682 OGs in *Bacillaceae *and *Enterobacteriaceae*, respectively (class CC: core to core), where the increase in the number of OGs in each set is primarily due to lineage-specific duplications in each family. On the other hand, 142 and 559 core OGs in *Bacillaceae *and *Enterobacteriaceae*, respectively, have some orthologs in the counterpart families that are not included in the core OG set (class CO: core to non-core ortholog), and 593 and 884 core OGs, respectively, are specific to each family (class CN: core to none). As expected, the majority of the essential genes (around 200 genes for each) are included in the CC class. To further examine the difference among these types, we studied the proportions of the KEGG functional categories (Figure 5B). The "core functional" characteristics of the core genes described above appear to be mainly linked to the CC class. In fact, most of the genes involved in primary metabolism and information processing are included in the CC class (Figure 5B; see also the KEGG map shown in Additional file 5). On the other hand, one prominent feature of the CN class in the *Bacillaceae *core is the larger proportion of the sporulation function, an obvious *Bacillus*-specific function. In the CN class, the majority of OGs are uncategorized, while in the CC class the proportion of uncategorized OGs is around 30%.

### Parameter dependency and robustness of the method

**Figure 6:**
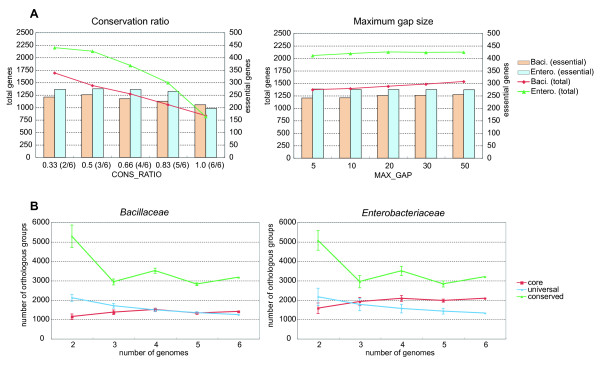
**Results of the parameter dependency test and the robustness test.** (A) The parameter dependency test. The total number of OGs (line, left axis) and the number of essential genes (bar, right axis) included in the core set generated from the *Bacillaceae *(red lines and peach bars) and *Enterobacteriaceae *(green lines and light blue bars) datasets are shown for each parameter value of the conservation ratio (left) and allowed gap size (right). (B) The robustness test. The average number of structural core (red), universally conserved (green) and ≥ 50% conserved (blue) OGs for each number of input genomes in the test datasets that are generated as subsets of the original dataset of *Bacillaceae *(left) and *Enterobacteriaceae *(right). The error bars show the standard deviations.

Our method of core structure extraction includes some arbitrary parameters, among which the two most important ones are *CONS_RATIO *and *MAX_GAP*, which define the degree of conservation required for core genes and the neighborhood of each gene, respectively (note that we set *NBR_CONS_RATIO *= *CONS_RATIO*). Here, we examined the dependency of these parameters on the results of the core extraction in terms of the total number of genes in the core set and the number of essential genes contained in the core set (Figure 6A).

When the *CONS_RATIO *parameter was tightened from 0.33 (2/6) to 1.0 (6/6), the number of the resulting core gene set decreased from 1690 to 834 in *Bacillaceae *and 2196 to 818 in *Enterobacteriaceae *(Figure 6A, left). Thus, the size of the core gene set can be greatly influenced by the *CONS_RATIO *parameter. On the other hand, the effect of the *MAX_GAP *parameter was relatively small (Figure 6A, right). The number of essential genes included in the core gene set generally increased as the number of core genes increased, but this number appeared to almost reach the maximal value with our default parameter set (*CONS_RATIO *= 0.5, *MAX_GAP *= 20). Thus, we can very roughly say that, by using our default parameter set, we were able to obtain the smallest core gene set that almost maximally covers the essential genes.

Next, we examined the robustness of the resulting core structures when changing the set of input genomes. In this test, we extracted six genomes for each family by choosing a representative genome from each set of closely related genomes (organisms with an asterisk in Table 1), and generated all the possible subsets containing two to five genomes. Then, using each of these subsets as the input genome, we ran the CoreAligner program to define the core structure and counted the number of resulting core OGs. We also examined the numbers of universal (*CONS_RATIO *= 1) and conserved (*CONS_RATIO *= 0.5) OGs (regardless of whether they belonged to the core or not) that were counted using the same subsets of genomes. Figure 6B shows the average number of the core, universal and conserved gene sets for each number of input genomes. As expected, the number of universal genes decreases monotonically as the number of genomes increases. In contrast, the number of conserved genes fluctuates widely with the change in the number of genomes, probably because the actual *CONS_RATIO *values fluctuate due to the rounding-up effect even when a constant cutoff value (0.5) is used. Curiously, the distributions of these average values appear to be very similar between *Bacillaceae *and *Enterobacteriaceae*, although the latter has much larger variances than the former. On the other hand, the number of core genes is quite different in the two families, as already mentioned in the previous sections. This clearly indicates that our criterion based on syntenic conservation defines a quite different set of core genes than the criterion based only on the presence of genes in each genome. Moreover, the number of core genes shows a relatively stable pattern in both families (Figure 6B). In fact, although similar fluctuations, probably due to the rounding-up effect, are again observed, the magnitude of fluctuation is much smaller than that for the conserved genes described above. These observations suggest that the use of synteny information with a relaxed conservation criterion (*CONS_RATIO *< 1) helps the CoreAligner program to identify robust and reliable core gene sets, although the setting of the *CONS_RATIO *parameter still remains somewhat arbitrary.

### G+C content of the third codon positions

Our main working hypothesis is that the core structure extracted here is mainly inherited through vertical transfers throughout evolution. Several methods have been developed for identifying horizontally transferred genes [4], and detecting anomalous nucleotide compositions is a common approach for identifying them using a target genomic sequence alone [36-40]. Among them, the G+C content of the third codon positions (GC3) is the most basic but still an effective characteristic, although the "amelioration" process unfolding in the course of genomic evolution may diminish its effectiveness [41]. Since it is known that highly expressed genes such as ribosomal proteins also have specifically biased codon usage that generally correlates with tRNA abundance [42], we predicted highly expressed genes based on the codon usage of ribosomal proteins [43] and eliminated them from the analysis.

**Figure 7:**
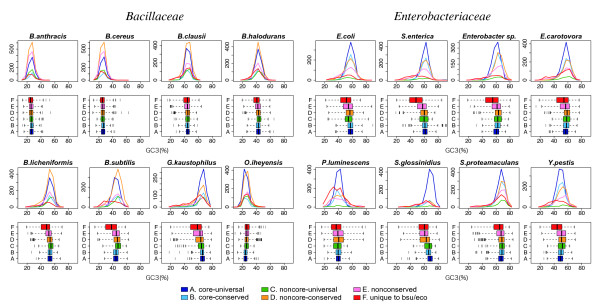
**Distribution of the GC3 percentages. **The distributions are shown for each of the six classes, A-F, as a histogram (upper) and a box plot (lower) for each genome. In each box plot, whiskers are extended at most 2.5 times the interquartile range, and outliers are indicated by vertical bars.

We calculated the GC3 values of the genes in each genome and examined their distribution for each of the six classes, A-F, defined above (Figure 7, upper). The GC3 values of the class-A genes (core-universal) show bell-shaped distributions with relatively sharp peaks. Similar distributions can be seen in classes B (core-conserved), C (noncore-universal) and D (noncore-conserved), but their shapes and sizes are different depending on the organism. The distributions of classes E (nonconserved) and F (unique) are generally broader and of a more irregular shape. The range of each distribution can be seen more clearly in the box plots (Figure 7, lower), which show that, generally, the core genes (A and B) are distributed within smaller ranges than the non-core genes. To examine the relationship in a more quantitative manner, we calculated the mean and the standard deviation for each distribution and compared the results (Additional file 6). We can confirm that the standard deviation generally increases in the order of A to F, and more specifically, that the relationship A, B < C, D < E, F holds in all of the 16 genomes, except that the latter inequality does not hold in the *S. glossinidius *genome where the number of non-core genes is extremely small. The smaller variance of the GC3 percentage of the core genes appears to support our working hypothesis that the core structure is generally indigenous to each genome.

From the box plots in Figure 7, we can see that there are some outliers in the core classes, and in some cases the number of outliers in the core classes is even larger than that of outliers in the non-core classes. However, we should notice that the number of genes and the distribution shapes are quite different among the classes, and thus a direct comparison of the number of outliers in these plots is meaningless. To understand this point more precisely, we identified the genes with significantly biased GC3 values using the condition |*x*-*μ*|/σ > 3.29, where *μ *and *σ *are the mean and the standard deviation, respectively, of the GC3 values of the core genes (classes A and B), and the cutoff value corresponded to *p *< 0.001 (for a single comparison) when normal distribution was assumed. Then, we compared the ratios of genes with biased GC3 values to the total number of genes between classes (Additional file 7). The result indicates that the ratios of the biased genes again generally increase in the order of A to F. Especially, the ratios are less than 1% in class A for all species and class B for most of the species, which is again consistent with our working hypothesis.

### Phylogenetic analysis

**Figure 8:**
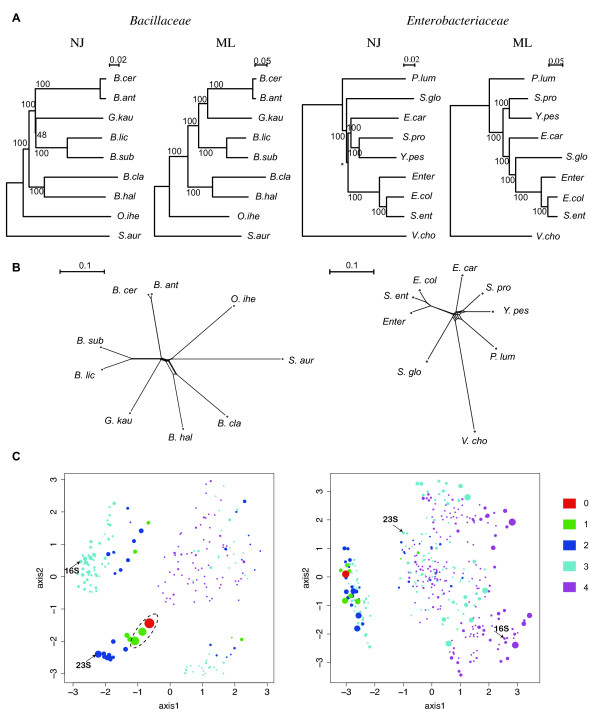
**Phylogenetic relationships among the core genes of *Bacillaceae *and *Enterobacteriaceae*.** (A) Phylogenetic trees of the concatenated core sequences that were constructed using the neighbor-joining (NJ) method (by CLUSTALW) and the maximum likelihood (ML) method (by MOLPHY). On the branch with an asterisk, the program did not assign any bootstrap value. (B) Phylogenetic networks of the concatenated core sequences constructed using the NeighborNet method (by SplitsTree). (C) Multidimensional scaling analysis of the phylogenetic tree topologies of the individual core genes. A tree topology is represented as a filled circle whose area is proportional to the number of occurrences and whose color indicates the distance from the ML tree topology of the concatenated core sequences (A), which itself is represented as a red circle. The topologies of the 16S and 23S rRNAs are also indicated by arrows.

If the core structure in each genome indeed shares the same evolutionary history, we can construct a very reliable phylogenetic tree by using a long concatenated sequence comprised of the core genes. For this purpose, we collected the universally conserved, one-to-one correspondence core OG sets comprising 712 and 1081 OGs for *Bacillaceae *and *Enterobacteriaceae*, respectively, and constructed phylogenetic trees using the concatenated alignments of the conserved blocks in these sequences comprising 199502 and 334889 residues, respectively. Using the neighbor-joining (NJ) method [44] and the maximum likelihood (ML) method [45], we were able to obtain highly resolved phylogenetic trees in which most of the branches, except some very short branches in the NJ trees, had bootstrap values of 100%, but the tree topologies constructed by different methods did not exactly coincide with each other (Figure 8A). In fact, such high confidence levels can be attained simply because of the extremely large sample sizes (number of alignment columns) and do not necessarily indicate the true reliability if there exists a systematic bias [46]. To examine whether the data indeed support tree-like phylogeny, we also constructed phylogenetic networks using the NeighborNet algorithm [47] (Figure 8B). The results showed that some internal branches exhibited a network-like (non-tree-like) structure that generally indicates a reticulate event such as horizontal transfer [48]. Especially, in the *Enterobacteriaceae *phylogeny, a network-like structure was found around the center of the phylogenetic radiation where the internal nodes were concentrated. Although the degree of incongruence appears not to be so large, this structure can obscure the phylogenetic relationship among organisms branching from there, and probably caused the above discrepancy of phylogeny.

To further investigate the congruence among the core gene phylogenies, we constructed ML trees of individual core OGs and compared them. As a result, we found 208 and 370 distinct topologies in the *Bacillaceae *and *Enterobacteriaceae *datasets, respectively, which are fairly large considering that there exist only 945 possible topologies among the 7 taxa examined here; the majority-rule consensus trees of these individual trees showed the same or a similar topology to the concatenated ML trees (Figure 8A) (data not shown). We visualized the set of the resulting tree topologies using multidimensional scaling (MDS) analysis based on the Robinson-Foulds topological distance [49] (Figure 8C). The results were again contrasting: there are three distinct major topologies that correspond to the alternative topologies of the ambiguous relationship in the *Bacillaceae *dataset (enclosed by the dotted ellipse in Figure 8C, left), whereas a more dispersed distribution can be observed in the *Enterobacteriaceae *dataset (Figure 8C, right). These observations clearly indicate that there is a severe limitation when inferring phylogeny using only a single or very few genes. The problem cannot be avoided even when using some "gold standard" genes; the phylogenetic topologies of the 16S and 23S rRNA sequences, the genes most conventionally used for inferring phylogeny, are located far from the representative topologies in our MDS plot (except *Bacillaceae *23S rRNA, which is fairly close to the representative one) (Figure 8C).

Unfortunately, the observed topology dispersion (Figure 8C) also appears to challenge our working hypothesis that all core genes share the same evolutionary history. However, we should consider the effect of the statistical fluctuation behind the variation of tree topology. To examine this, we performed a Shimodaira-Hasegawa (SH) test [50] for each of the core OGs and estimated the number of OGs whose individual ML tree topologies are significantly different from that of the representative genomic tree topology taken from the ML tree of the concatenated core sequences (see Figure 8A). In this test, we also performed the same test using the non-core OGs that have a one-to-one correspondence; here, we considered non-universal OGs as well as universal ones because there are only a few universal non-core OGs, and summed up the results for each of the effective number of genomes (*N*) contained in the OGs (Table 3). When using a significance level of 5%, we found that 4.3% (32/739) and 4.1% (45/1099) of the core OGs of *Bacillaceae *and *Enterobacteriaceae*, respectively, with *N *= 7 had significantly different topologies from the representative one (Table 3). In other words, the observed number of significantly different cases was within the expected value (5%). Therefore, the number of incongruent cases may not be as large as it appears to be in Figure 8C. In contrast, in total, around 20% of the tested non-core OGs had significantly different topologies at the 5% level (Table 3). Fisher's exact test showed that the number of incongruent cases of the non-core OGs was significantly larger than that of the core OGs, except in the case of *N *= 7 in *Enterobacteriaceae*, where the sample size of the non-core cases was extremely small (Table 3). Therefore, we can conclude that the core genes indeed have a greater tendency to exhibit congruent phylogenies, at least in comparison with the non-core genes. Note that a greater congruency of the core than that of the non-core is observed in virtually every group of effective number of genomes (*N*), indicating that the issue of whether it is the core or not is more directly connected to phylogenetic congruency than the universality of genes. Note also that the dataset used to construct the reference tree of this test is included in the cell of (*N *= 7, core) in Table 3, which means that the comparison at *N *= 7 may be somewhat unfair but the comparisons at other *N *are not.

**Table 3:** The number of incongruent topologies detected by the Shimodaira-Hasegawa (SH) test.

*Bacillaceae*					
	core		non-core		
*N*	rejected	total	rejected	total	*p*-value

4	3 (3.5)	85	32 (14.0)	228	0.004573
5	11 (6.2)	176	38 (21.6)	176	2.170e-05
6	17 (5.4)	314	34 (29.6)	115	1.899e-10
7	32 (4.3)	739	9 (15.5)	58	0.001705

total	63 (4.8)	1314	113 (19.6)	577	<2.2e-16

*Enterobacteriaceae*					

	core		non-core		
*N*	rejected	total	rejected	total	*p*-value

4	6 (5.0)	121	19 (17.1)	111	0.002496
5	12 (5.1)	235	21 (24.4)	86	2.784e-06
6	28 (5.7)	490	9 (34.6)	26	2.431e-05
7	45 (4.1)	1099	0 (0.0)	8	1

total	91 (4.7)	1945	49 (21.2)	231	6.409e-16

The SH test was performed for all OGs (both core and non-core) that have a one-to-one correspondence using the TREE-PUZZLE program with the ML topology (or its subtree) of the concatenated core sequences (Figure 8A) as a reference tree, and the numbers of rejected (significantly incongruent) cases were compared between core and non-core classes using the Fisher's exact test. The first column (*N*) indicates effective number of genomes where genomes of closely related species were counted only once. The column "rejected" indicates the number of cases rejected by the SH test at a significance level of 0.05; the number in parentheses is the percentage of rejected cases. The last column indicates the *p*-value of a one-sided Fisher's exact test.

## Discussion

In this work, we developed a method for identifying the core structure among moderately related microbial genomes by constructing a genome alignment based on the consensus gene order. We applied the method to the genome sets of the families *Bacillaceae *and *Enterobacteriaceae *and characterized the resulting core structures in terms of gene function, essentiality, nucleotide content and phylogenetic relationship. The results showed that the core structures covered functionally important genes (Figure 4 and Figure 5), including most of the essential genes (Additional file 4), and generally had more homogeneous GC3 values (Figure 7) and phylogenetic tree topologies (Table 3) than the non-core genes. All of these characteristics are to be expected in light of the core genome concept. We also examined the parameter dependency and robustness of our method, and found that it can offer a more robust core genome definition than simpler approaches that consider only universality or a fixed conservation ratio as the criterion (Figure 6B). This robustness enabled us to define plausible core structures even when some of the genomes have degenerated due to changes in environmental conditions such as those arising from symbiosis (Table 2) that have made the existence of certain core genes unnecessary (as long as the ratio of degenerate genomes is sufficiently smaller than the *CONS_RATIO *parameter; actually, it is probably better to eliminate extremely small genomes to avoid unexpected effects). On the other hand, our method is dependent on the conservation ratio parameter (Figure 6A). Although we believe that the parameter setting used here is better for defining plausible core gene sets in terms of coverage of the core functions for both *Bacillaceae *and *Enterobacteriaceae*, we might need to use different parameters when we consider different taxonomic groups. In fact, it might be better to use more relaxed conditions when we analyze genomes whose core structure is subject to substantial disruption. Another related issue is the size of the core, which is likely to decrease when the diversity of the group for which the core is determined increases. Here, we showed that the core of *Enterobacteriaceae *(2125 genes) is larger than that of *Bacillaceae *(1438 genes), and that *Bacillaceae *is indeed more diverse than *Enterobacteriaceae*, at least in terms of sequence diversity (see Additional file 1, upper right and Figs. 8A and 8B). However, the relative frequencies of genomic rearrangement and nucleotide mutation may be different among different lineages, and to investigate this issue in a more quantitative manner, we need to extend the analysis to a broader range of taxonomic groups. Applying our method to more varied taxonomic groups is an important future task.

In this work, we used the coverage of the essential genes as an indicator for evaluating the core gene sets. Consequently, most of the functionally important genes are included in the resulting core gene sets, especially in the intersection of the two core gene sets (Figure 5). On the other hand, the resulting core gene sets also include substantial uncharacterized genes, and many of the taxon-specific core genes are still functionally uncharacterized (see Figure 5, but note that the genes to which a KEGG category is assigned are not necessarily uncharacterized). It is natural to think that such taxon-specific core genes have some important but to date uncharacterized functions that are related to taxon-specific features, since they are likely to have been inherited in that taxon throughout evolution, although we generally need more extensive analysis with a greater variety of genomes to claim taxon specificity. However, we should also note that the significance of the gene order conservation of individual core genes can vary from gene to gene, and that some of them could be less important but co-inherited simply as "hitchhikers."

The rationale behind our definition of "core genome" as an indigenous, vertically transmitted structure based on syntenic conservation is that horizontally transferred genes are unlikely to insert themselves at the same chromosomal position. Although both the GC3 analysis and the phylogenetic analysis generally support the hypothesis that the extracted core genes are indigenous and share the same evolutionary history, there are some exceptional cases. For example, several genes with extremely biased GC3 values were found in the core genes (Figure 7) and some core genes appear to have significantly different phylogenetic tree topologies from that of the concatenated core sequences (Figure 8 and Table 3). Although the SH test showed that the number of incongruent cases may not be as large as it appears to be (Table 3), it should be noted that the SH test has been suggested to be too conservative [12,51].

**Figure 9:**
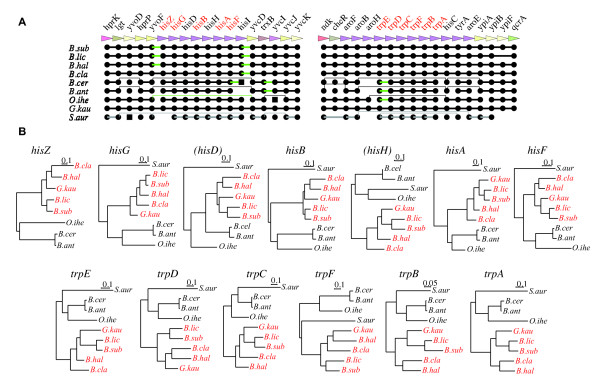
**Examples of cases where phylogenetic incongruence and the local rearrangement of the core structure are linked to each other.** (A) The core genome alignment of *Bacillaceae *around the histidine and tryptophan biosynthetic operons. The names of the genes showing significant phylogenetic incongruence are shown in red. (B) The ML trees of the genes in these operons. The genes that were not detected as incongruent by the SH test are enclosed in parentheses. The names of the organisms in which the core arrangement around the operon are conserved are shown in red. Note that these trees are unrooted because outgroup rooting is not possible here due to the possible existence of horizontal transfers.

Actually, we used a relaxed criterion for syntenic conservation (*CONS_RATIO *< 1) in our core genome alignment procedure. Therefore, some of the exceptional cases could have arisen due to this relaxed criterion. Notable examples are shown in Figure 9, where two possible operons related to the histidine and tryptophan biosynthesis pathways, respectively, contain genes with significantly incongruent phylogeny identified by the SH test (Table 3). In both cases, the operonic structures are apparently translocated in the *B. anthracis, B. cereus *and *O. iheyensis *genomes from the conserved core arrangement (Figure 9A), and genes in the same set of genomes also form a cluster distinct from the other *Bacillaceae *genes in the phylogenetic tree of each family (Figure 9B), suggesting that these operonic structures were likely to be replaced with horizontally acquired ones in these organisms. Although only a limited number of significantly incongruent cases were found in the core by the SH test here, this phenomenon might be more common because we can find similar small local rearrangements in the resulting core structure (see Additional file 2). Nevertheless, we dot not consider it a serious problem, since it is an expected consequence of our definition of the core, where genes are inherited "mainly" rather than "exclusively" through vertical transfer. On the other hand, a more serious concern may arise if the gene order in the resulting core structure is only very poorly conserved, especially when one uses too large a *MAX_GAP*: the similarity of the relative gene location rather than the conservation of the exact gene order might be a result of convergent evolution, which is not what we expect. Although we think that the resulting core structures presented here are so well-ordered that they are unlikely to suffer from this problem, in order to avoid the problem in more general cases, we may need to introduce some solution, such as a statistical measure, to give guideline to users who must choose appropriate parameter values.

In addition to the problems arising from the relaxed conditions, there are more fundamental exceptions to the above rationale. One such exception occurs when horizontally transmitted segments insert themselves into the same (orthologous) target sites. In fact, we encountered such a case during the experimental construction of the *Enterobacteriaceae *core structure (Additional file 8), where the region is conserved in only four genomes, but in three of the four cases the region is located at the same site (adjacent to the *yjdC *gene). This region in the *S. enterica *genome indeed corresponds to the genomic island named "Stic134F" defined in the Islander database [52], and there are Phe-tRNA genes at both ends of each of these regions, which are likely to have been a common integration site of this island. We eliminated these regions by introducing a procedure to identify locally non-conserved regions in each genome (see Method, step 5). However, the current procedure depends on the somewhat *ad hoc *assumption that inserted segments exceed a certain length. Therefore, although we believe that the procedure worked at least for the dataset of this study, additional evidence, such as genes (or pseudogenes) or repetitive elements related to some mobile elements, might be needed to correctly identify such regions in more general cases, and one may need other tools (e.g., [53]) for such tasks.

Homologous recombination is another factor that can disrupt the evolutionary homogeneity of the core genes defined through synteny. Although the importance of recombination within a bacterial population has been demonstrated in several species [54,55], direct genetic exchange among the genomes compared here is very unlikely, because here we compared only moderately related genomes where nucleotide identities between orthologs are in many cases only 60–70% and hence there is little chance of having the sufficiently long identical segments required for successful homologous recombination [56-59]. However, we cannot exclude the possibility that recombination occurred frequently during a certain period after speciation, which might explain the observed network-like phylogeny (Figure 8B). In addition, a successive chain of recombination events between closely related sequences could result in genetic exchange between more distantly related genomes, and the recombination rate might have been elevated by inactivation followed by the reacquisition of mismatch repair proteins, as suggested for *E. coli *strains [60]. On the other hand, there are some exceptions to the above nucleotide identity range, among which the most notable ones are the rRNA genes; the identities between the rRNA genes are generally more than 90%. This might give a plausible explanation why the topologies of the rRNA gene trees are so different from those of the concatenated trees (Figure 8C), as pointed out previously [8,55]. In any case, the possibility of homologous recombination across a species boundary, if any, should be an inherent feature of prokaryotic genome evolution, and could have been involved in the core genome formation. Therefore, the extraction of core structures among genomes at various levels of relatedness can provide a basis for further studies of this phenomenon.

Although we have focused on the problem of identifying the genomic core among moderately related prokaryotic genomes throughout this work, our genome alignment method itself does not assume any particular prokaryote-specific features, and thus, in principle, can be applied to more general purposes, including eukaryotic genome comparison. There have been numerous studies on multiple genome comparison, which we think can be primarily classified into the following categories: (1) nucleotide sequence alignment among closely related genomes [61-64]; (2) gene order comparison among moderately related genomes for investigating the genomic rearrangement history [65-67]; (3) finding conserved gene clusters among distantly related genomes for identifying operons and über-operons [27,68,69]. At first glance, the problem of the present study may appear to be most similar to (2). In fact, the problem of finding consensus gene orders considered here can also be formulated as a median breakpoint problem assuming a star-like phylogeny [65]. However, here we used a strategy similar to that of Rogozin *et al*. [27], mainly because the core genome identification problem has a "local alignment"-like nature, in that its goal is to extract well-conserved genomic segments, in contrast to the general median problem whose goal is to find a consensus order that spans the entire genome. We think that our approach has a practical advantage especially for the bacterial core genome extraction described here, but possibly also for eukaryotic genome comparison when well-conserved regions are limited. One drawback of our current approach is that it assumes star-like phylogeny and cannot treat hierarchical phylogenetic relationships among genomes. One way of incorporating such relationships is to introduce a weighting scheme, which has been investigated in the field of multiple sequence alignment (e.g., [70]). Another more interesting approach is to consider the evolutionary scenario more explicitly when constructing an alignment (e.g., [71]). In any case, the incorporation of phylogenetic relationships is crucial for investigating the evolution (formation or erosion) of the core structure, which is an important next step toward understanding prokaryotic genome evolution.

## Conclusion

The present study demonstrates that the procedure (CoreAligner) for multiple genome alignment based on gene order conservation can provide an effective approach to identify the genomic core among moderately related microbial genomes. Identifying the genomic cores among various taxonomic groups will provide a basis for further comparative studies utilizing the rapidly accumulating genomic data for understanding microbial diversity and evolution.

## Methods

### Preparation of genomic data

We used the Microbial Genome Database for Comparative Analysis (MBGD) [14,72] in order to construct the orthologous groups (OGs) of the specified sets of related genomes. In this work, we used two sets of genomes belonging to the families *Bacillaceae *and *Enterobacteriaceae *(Table 1), as per the NCBI Taxonomy database for taxonomic classification.

### Construction of a core genome alignment by ordering the OGs

**Figure 10:**
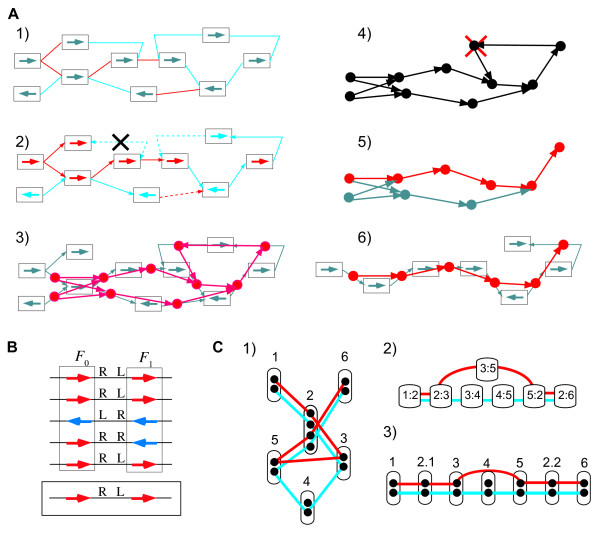
**The procedure for constructing a core genome alignment.** (A) Overview of the procedure, which consists of the following steps: (1) Initial conserved neighborhood graph. The color of each edge (red or blue) indicates that two genes incident to it are in the same or opposite directions, respectively. (2) Assignment of a consistent orientation based on the MST. The edges included in the MST are drawn with sold lines, and other edges are drawn with broken lines. The node colors represent node directions, Dir(*F*). The edges are directed according to the resulting orientation, except the edge indicated by the X mark that cannot be assigned a consistent orientation; vertices incident to a blue edge should be in opposite directions (assigned different colors) in a consistent orientation. (3) Conversion of the original graph (gray) to the triplet graph (red). (4) Elimination of loops. The eliminated vertex is indicated by the X mark. (5) Identification of the maximum path of the graph. The maximum path is indicated in red. (6) Restoration of the original graph and construction of the genome alignment. (B) Determination of the representative relative direction between two neighboring OGs, *F*_0 _and *F*_1_, by majority vote. (C) An example of conversion of an original neighborhood graph to a triplet graph. (1) A neighborhood graph comprising six nodes (OGs) and seven edges. The red and blue lines indicate the two genomes used to construct the graph. Node 2 contains two (in)paralogous lineages that cause loop formation. (2) A triplet graph resulting from the conversion of graph (1). Note that the graph contains no loop. (3) A neighborhood graph resulting from a conversion back from the triplet graph (2). Note that node 2 is divided into two nodes.

Our procedure, CoreAligner, for constructing an alignment of the core structure basically consists of finding the best conserved ordering of a pre-identified set of OGs. To construct the OGs, we used the DomClust algorithm implemented on the MBGD server, which can handle both inparalogous relationships and domain fusion/fission events in the process of ortholog grouping [24], although, in principle, other programs or databases can also be used for this purpose. Figure 10A gives an overview of the CoreAligner procedure, which consists of the following steps: 1) extraction of the conserved neighborhood relationships and construction of an initial conserved neighborhood graph, *G*_0_; 2) assignment of a consistent orientation based on the minimum spanning tree of *G*_0 _and construction of a directed graph, *G*_1_; 3) conversion of *G*_1 _into a triplet graph, *G*_2_, in which each edge represents the arrangement of three genes rather than two genes; 4) elimination of the loops from *G*_2 _and construction of a directed acyclic graph, *G*_3_; 5) identification of the paths of maximum weight on *G*_3 _using the dynamic programming algorithm; 6) construction of an alignment by restoring the original graph. In the following, we describe the procedure step by step.

#### Step 0: Extraction of the conserved OGs

Before the procedure, we retained only those OGs that are conserved among at least a given ratio (*CONS_RATIO*) of the total number of genomes. Throughout this procedure, the genomes that are close enough to each other are grouped so that the genomes in each group are counted only once in order to avoid bias; here, we used the species groups shown in Table 1, which were determined through visual inspection of the pairwise comparison data (see Additional file 1). Let **F **denote the resulting set of conserved OGs, and let **G **be the set of genomes compared. We consider each genome, *G *∈ **G**, as an ordered gene list, *L*_*G *_= [*g*_1_, *g*_2_,..., g_*N*_], where each gene, *g*_*i*_, in *L*_*G *_is ordered according to the genomic position on *G *and belongs to one of the conserved OGs, *F*_*j *_∈ **F**. Let order_*G*_(*g*) denote the ordinal position of the gene, *g*, in the list *L*_*G*_. We define the distance between genes *g*_1 _and *g*_2 _on the circular genome, *G*, as Dist(*g*_1_, *g*_2_) = min [Diff(*g*_1_, *g*_2_),| *L*_*G*_|-Diff(*g*_1_, *g*_2_)], where Diff(*g*_1_, *g*_2_) = |order_*G*_(*g*_1_)-order_*G*_(*g*_2_)|.

#### Step 1: Generation of the initial conserved neighborhood graph

In this step, we extract the OG pairs that are located within *MAX_GAP *genes in at least *NBR_CONS_RATIO *(= *CONS_RATIO*, by default) of the total number of genomes and construct a conserved neighborhood graph. First, let us define a neighboring OG pair. A gene pair, *g*_0_, *g*_1 _∈ *G*, is a neighboring gene pair if *Dist*(*g*_0_, *g*_1_) ≤ *MAX*_*GAP*, and an OG pair, *F*_0_, *F*_1 _∈ **F**, is a neighboring OG pair if there exists a neighboring gene pair *g*_0_, *g*_1 _such that *g*_0 _∈ *F*_0_, *g*_1 _∈ *F*_1_. Let *Ng*(*F*_0_, *F*_1_) denote the set of neighboring gene pairs between *F*_0 _and *F*_1_. We also consider a relative direction for each neighborhood OG pair. The relative direction between two neighboring genes, denoted by Rdir(*g*_0_, *g*_1_), is either (L, L), (L, R), (R, L) or (R, R), where L and R signify the left and right end of each gene, respectively, and the relative direction between two neighboring OGs, denoted by Rdir(*F*_0_, *F*_1_), is determined by majority vote among (*g*_0_, *g*_1_) ∈ *Ng*(*F*_0_, *F*_1_) (Figure 10B). In this majority vote, each neighboring gene pair is weighted as a function of the reciprocal of distance, *weight*(*g*_0_, *g*_1_) = [1/*Dist*(*g*_0_, *g*_1_)]^*α*^, with the parameter *α*. Throughout this work, we simply set *α *= 1.

Let *c *be the number of neighboring gene pairs in the majority direction, *i.e*., |{(*g*_0_, *g*_1_)|Rdir(*g*_0_, *g*_1_) = Rdir(*F*_0_, *F*_1_)}|. The pair (*F*_0_, *F*_1_) is considered to be a conserved OG pair if *c *satisfies the conservation criterion (*c*/*N *≥ *NBR*_*CONS*_*RATIO*)). Among the conserved OG pairs, we further extract the proximal OG pairs, which are defined as follows: a conserved OG pair *F*_0_, *F*_1 _is a proximal conserved OG pair if there exists a pair of neighboring genes, *g*_0 _∈ *F*_0_, *g*_1 _∈ *F*_1_, between which there is no gene *g*_2 _∈ *F*_2_, such that either (*F*_0_, *F*_2_) or (*F*_2_, *F*_1_) is a conserved OG pair. Finally, the conserved neighborhood graph, *G*_0 _= (*V*_0_, *E*_0_), is constructed with vertices *V*_0 _= **F **and edges *E*_0 _⊆ *V*_0 _× *V*_0 _that are the set of proximal conserved OG pairs. On each edge, an edge weight is assigned as the sum of the weights of the neighboring gene pairs: $Weight(F_{0},F_{1})=\sum_{g_{0},g_{1}\in Ng(F_{0},F_{1})}1/Dist(g_{0},g_{1})$.

#### Step 2: Conversion to a directed graph (direction assignment)

In this step, we construct a minimum spanning tree (MST), *T*_*M*_, of the conserved neighboring graph, *G*_0_, and assign a consistent orientation to each vertex and edge based on the relative direction on each edge of the tree, *T*_*M*_; thus the procedure converts the undirected graph, *G*_0_, into a directed graph, *G*_1_. To construct the MST, a weighted distance is assigned to each edge (*F*_0_, *F*_1_) as Wdist(*F*_0_, *F*_1_) = 1/Weight(*F*_0_, *F*_1_), so that highly weighted edges are likely to be incorporated into the MST. For each *F *∈ **F**, a vertex orientation, Dir(*F*) = {1, -1}, is determined based on the MST *T*_*M *_as follows: starting from an arbitrary vertex, *F*_0 _∈ **F**, for which we set Dir(*F*_0_) = 1, the orientation is determined along each edge, (*F*_*i*_, *F*_*j*_) ∈ *T*_*M*_, by Dir(*F*_*j*_) = Dir(*F*_*i*_) × sgn [Rdir(*F*_*i*_, *F*_*j*_)], where sgn(*rdir*) = 1 if *rdir *∈ {(*R*, *L*), (*L*, *R*)} and sgn(*rdir*) = -1 otherwise (see Figure 10B; in Figure 10A2, the node colors and edge colors represent Dir(*F*_*i*_) and sgn [Rdir(*F*_*i*_, *F*_*j*_)], respectively, where red = 1 and blue = -1). In addition, the orientation of each edge, (*F*_*i*_, *F*_*j*_) ∈ *T*_*M*_, is determined by Dir(*F*_*i*_, *F*_*j*_) = Dir(*F*_*i*_) × side(*F*_*i*_, *F*_*j*_), where side(*F*_*i*_, *F*_*j*_) = 1 if *F*_*j *_is on the right side of *F*_*i *_and side(*F*_*i*_, *F*_*j*_) = -1 otherwise (Dir(*F*_*i*_, *F*_*j*_) is represented by an arrow in Figure 10A2). Here, the edge orientation represents the consensus gene order, and the node orientation represents the consensus gene orientation along the virtual genome. The orientation can be consistently assigned as long as it is done along a tree. In addition, for each remaining edge, (*F*_*i*_, *F*_*j*_) ∈ {*e*|*e *∈ *E*_0_, *e *∉ *T*_*M*_} (broken lines in Figure 10A2), the edge orientation is calculated by the same formula, Dir(*F*_*i*_, *F*_*j*_) = Dir(*F*_*i*_) × side (*F*_*i*_, *F*_*j*_), but the orientation is assigned only when the values calculated from both directions is consistent, *i.e.*, Dir(*F*_*i*_, *F*_*j*_) = -Dir(*F*_*j*_, *F*_*i*_); otherwise, the edge is removed from the converted graph, *G*_1 _(X mark in Figure 10A2). Finally, a directed graph, *G*_1 _= (*V*_1_, *E*_1_), with vertices *V*_1 _= **F **and edges *E*_1 _⊆ *V*_1 _× *V*_1 _is constructed by connecting the vertices from *F*_*i *_to *F*_*j *_if Dir(*F*_*i*_, *F*_*j*_) = 1 and from *F*_*j *_to *F*_*i *_if Dir(*F*_*i*_, *F*_*j*_) = -1.

#### Step 3: Conversion to a triplet graph

In this step, the directed graph, *G*_1_, is converted into a triplet graph, *G*_2_, in which each edge represents the order of three OGs (*F*_*i*_, *F*_*j*_, *F*_*k*_), rather than two OGs (*F*_*i*_, *F*_*j*_), as in each edge of the graph *G*_1_. For this purpose, a neighborhood OG list, *Nf*(*g*, *side*) = {*F*'|*Dist*(*g*, *g*') ≤ *MAX*_*GAP*, side(*g*, *g*') = side, *g*' ∈ *F*'}, is prepared for each *g *∈ *F *and side ∈ {-1,1} (-1 and 1 signify the left and right sides, respectively). To convert *G*_1 _= (*V*_1_, *E*_1_) into *G*_2 _= (*V*_2_, *E*_2_), we set *V*_2 _= *E*_1_, and connect each pair of edges in *G*_1 _incident to a node, *F*_*j *_∈ *V*_1_, *i.e*., (*F*_*i*_, *F*_*j*_), (*F*_*j*_, *F*_*k*_) ∈ *E*_1 _= *V*_2_, in *G*_2 _in this direction if there exist some genes, *g *∈ *F*_*j*_, such that *F*_*i *_∈ *Nf*(*g*,-1) ∧ *F*_*k *_∈ *Nf*(*g*,1). This conversion guarantees that the triplet order (*F*_*i*_, *F*_*j*_, *F*_*k*_) represented by each edge of the graph *G*_2 _will actually appear in at least some genomes (the parameter *NBR_CONS_NUM2*; default 2). Figure 10C shows a simple example illustrating the effect of this conversion. Here, the OG represented by node 2 contains two inparalogous subgroups, due to which the graph contains a loop (Figure 10C1). However, the triplet graph conversion linearizes the graph (Figure 10C2), since the path of 5-2-3 in the original graph, which is responsible for the loop formation, does not exist in the actual sequences.

#### Step 4: Conversion to a DAG (loop elimination)

Although the triplet graph conversion at the previous step can eliminate some trivial loops as described above, generally there still remain loops in the directed graph, *G*_2_. In this step, we eliminate all loops by removing some vertices from the graph *G*_2 _(corresponding to the edges of the graph *G*_1_) to make a directed acyclic graph (DAG), *G*_3_. Since the problem of finding the minimum number of vertices to cut in a directed graph required for making a DAG (the minimum feedback vertex set problem) is NP-hard, we used a heuristic method called a "contraction algorithm" [73], which returns a correct answer when the graph is completely contractible through a series of contraction operations defined in the algorithm; otherwise, the algorithm still returns a valid answer within a reasonable time period, although its optimality is not guaranteed. Fortunately, it turned out that all cases treated here were contractible and hence the algorithm could solve the problem correctly. We conjecture that the graph *G*_2 _is contractible in most cases if the compared genomes are sufficiently close.

#### Step 5: Extraction of the set of paths of maximum weight

In this step, we find the longest paths in the DAG, *G*_3 _= (*V*_3_, *E*_2_), using a DP algorithm. For this purpose, we assign a weight to each vertex, *v *∈ *V*_3 _= *E*_1_, which actually corresponds to a neighboring relationship (*F*_*i*_, *F*_*j*_), as Weight(*v*) = Weight(*F*_*i*_, *F*_*j*_). The basic recursion formula for finding the path of maximum weight is as follows:

$$\mathrm{TotalWeight}(v_{m})=\underset{v_{n}\in \mathrm{Out}(v_{m})}{\max}(\mathrm{TotalWeight}(v_{n})+\mathrm{Weight}(v_{m})),$$

where *v*_*n*_, *v*_*m*_∈ *V*_3 _and Out(*v*_*m*_) is the set of vertices in which the out-edges of *v*_*m *_enter. The pair of vertices in the above formula is saved as an arc (*v*_*m*_, *v*_*n*_) of a directed graph (a set of trees, or forest), *T*_*P*_, which can be backtracked to find the path of maximum weight. The extracted maximum path, which we here call a "cluster," is added to the core structure if it meets the following criteria: (1) it contains at least a given number of OGs (the parameter *MIN_CLUSTER*; default 10), and (2) at least a given proportion of OGs are present in each genome (the parameter *SP_COVER*; default 0.5). The latter criterion is required to eliminate lineage-specific clusters, which are likely to belong to phages or genomic islands rather than the genomic core.

In some cases, a "local version" of this latter criterion is needed to eliminate problematical cases. In fact, during experimental applications of the CoreAligner procedure to the *Enterobacteriaceae *dataset, we encountered such a case where an apparent genomic island was included in the core structure (Additional file 8; see also Discussion). To eliminate such regions, we first identified locally sparse regions on each genome by finding a maximal scoring segment with a scoring system of absence = +1 and presence = -1, and if this score exceeded a certain cutoff point (here we used 20) in at least one genome, the resulting region was subjected to the test with the above *SP_COVER *criterion to determine whether the region should be removed or not.

To find out the path of the next maximum weight, we used a similar strategy to that for finding non-intersecting suboptimal sequence alignments [74]: each vertex of the maximum path is marked as deleted and assigned a bad score (-∞), and all of its descents are once removed from *T*_*P*_. Step 5 is repeated and the scores of the removed nodes are recalculated by DP and *T*_*P *_is reconstructed to find the next maximum path. The procedure is repeated until no (undeleted) vertex remains.

#### Step 6: Construction of an alignment from the paths of maximum weight

The set of paths extracted in the previous step is actually a triplet graph, which should be converted back to the original graph. This conversion can be done straightforwardly: for example, a path of 1:2-2:3 can be simply converted into 1-2-3. However, an interesting situation arises when an OG contains some inparalogous lineages, as in the example in Figure 10C, which may cause multiple appearances of the same node in the alignment path. Even in such a case, a genuine ortholog can typically be found among the inparalogs by examining the context of the alignment path (nodes 2.1 and 2.2 in Figure 10C3). To solve this problem, CoreAligner scores each gene according to the matching of the local context around it, and takes the best-scoring gene as a genuine ortholog. Here, the weight of the gene, *g*_0 _∈ *F*_0_, is defined as $\sum_{g_{1}\in F_{1}}1/\sqrt{Dist_{G}(g_{0},g_{1})Dist_{C}(F_{0},F_{1})}$, where Dist_*G *_and Dist_*C *_are the distances along the genome, *G*, that contains *g*_0 _and *g*_1 _and the consensus alignment, *C*, respectively, and the summation is taken over all *g*_1 _∈ *F*_1 _such that *Dist*_*G*_(*g*_0_, *g*_1_) ≤ *MAX*_*GAP *and *Dist*_*C*_(*F*_0_, *F*_1_) ≤ *MAX*_*GAP*.

### Identification of orthologs between *Bacillaceae *and *Enterobacteriaceae*

To examine the correspondence between the core gene sets of *Bacillaceae *and *Enterobacteriaceae*, we generated the OGs of the combined dataset of these two families by the DomClust program. Each OG of an individual family, say *F*_*B *_of *Bacillaceae*, is considered to correspond to an OG of the combined dataset, say *F*_*C*_, if more than half of the members of *F*_*B *_are included in *F*_*C*_. Then, we considered that two OGs of *Bacillaceae *and *Enterobacteriaceae*, say *F*_*B*_and *F*_*E*_, respectively, correspond to each other if both *F*_*B *_and *F*_*E *_correspond to the same OG of the combined dataset, say *F*_*C*_.

### Essential gene analysis

The sets of *B. subtilis *essential genes (271 genes) [32] and *E. coli *essential genes (300 genes) [33] were taken from the original papers. Three genes (*yabQ*, *yafF*, *yibJ*) were eliminated from the *E. coli *set, since those ORFs are treated as pseudogenes in the current RefSeq database. For the *E. coli *essential gene set, we also referred to the essential gene set of Kato *et al*. (302 genes) [35] obtained from the PEC database [75]. By taking the intersection of these two data sets, we obtained a more rigorous essential gene set containing 261 genes.

### G+C content of the third codon positions (GC3)

For the calculation of the GC3 values, we used only genes comprising more than 100 codons. We also eliminated from the dataset putative highly expressed (PHX) genes that have specifically biased codon usage patterns. Here, we used a simplified version of the previously proposed procedure to define the PHX genes [43]: a gene, *g*, is considered to be a PHX gene if the predicted expression level of *g *defined as *E*_*RP *_(*g*) = *B*(*g*|*All*)/*B*(*g*|*RP*) exceeds 1.05, where *B*(*g*|*G*) is the codon usage difference of *g *relative to the given gene set, *G*, according to Karlin and Mrázek [43], and *All *and *RP *denote the sets of all genes and ribosomal proteins in a given genome, respectively. This procedure eliminated around 150–300 genes for each genome in our dataset as PHX genes.

### Phylogenetic analysis

To simplify the phylogenetic analysis, we used only OGs that have a one-to-one correspondence (*i.e*., those containing neither duplication nor domain fusion/fission events). For each OG, a multiple sequence alignment was generated by CLUSTALW [76], and from that alignment the conserved alignment blocks suitable for phylogenetic analysis were extracted by the Gblocks program [77]. In order to create the phylogenetic trees of the core structure, we constructed a concatenated sequence alignment of the universally conserved one-to-one core OGs, and created a neighbor joining tree [44] using the CLUSTALW program and a maximum likelihood tree using the MOLPHY program [45] under the JTT model [78]. In addition, we created a phylogenetic network using the Neighbor-Net method [47] implemented in the SplitsTree program [48].

We also conducted phylogenetic analyses for individual core genes; here, maximum likelihood tree construction and the Shimodaira-Hasegawa test were performed using the TREE-PUZZLE program [79] under the JTT model. In this test, the relationships among the closely related species shown in Table 1 were fixed, and all the remaining possible topologies (maximally 945 topologies among the 7 taxa) were generated and tested. Here, we considered the ML topology of the concatenated core sequences (Figure 8A), or an appropriate subtree of it when considering a non-universal OG, as a representative genomic tree, and compared it with the ML topology of the individual OG.

Statistical analyses, including classical multidimensional scaling and Fisher's exact test, were performed using the cmdscale and fisher.test commands in the R package. The Robinson-Foulds topological distances [49] were calculated using the treedist command in the PHYLIP package [80]. For simplicity, we used the value of the resulting symmetric difference (which is always an even number) divided by 2.

## Abbreviations

OG: orthologous group; HGT: horizontal gene transfer; DP: dynamic programming; ORF: open reading frame; GC3: G+C content of the third codon positions; NJ: neighbor joining; ML: maximum likelihood; MDS: multidimensional scaling; SH test: Shimodaira-Hasegawa test; MBGD: Microbial Genome Database for Comparative Analysis; MST: minimum spanning tree; DAG: directed acyclic graph; PHX gene: putative highly expressed gene.

## Authors' contributions

IU carried out every substantial work in this study and approved the final manuscript.

## Supplementary Material

Additional file 1 **Pairwise comparisons between the organisms used in this study.** Click here for file

Additional file 2 **Complete figures of the core genome alignments.** Click here for file

Additional file 3 **Detailed lists of the structural core gene sets.** Click here for file

Additional file 4 **Essential genes identified in the *B. subtilis *and *E.coli *genomes.** Click here for file

Additional file 5 **Global metabolic map displaying the common core genes shared between *Bacillaceae *and *Enterobacteriaceae *drawn by the KEGG Atlas system.** Click here for file

Additional file 6 **Means and standard deviations of the GC3(%) values in each class.** Click here for file

Additional file 7 **Proportions of genes with significantly deviated GC3 values in each class.** Click here for file

Additional file 8 **A problematical case found during an experimental core structure extraction from the *Enterobacteriaceae *dataset.** Click here for file
