# Supplementary material for: Multiple genome alignment for identifying the core structure among moderately related microbial genomes
Source: BMC Genomics. 2008 Oct 31;9:515. doi: 10.1186/1471-2164-9-515 (PMC2615449; doi:10.1186/1471-2164-9-515)
Supplement: Additional file 1 — Pairwise comparisons between the organisms used in this study. [file 1471-2164-9-515-S1.pdf]

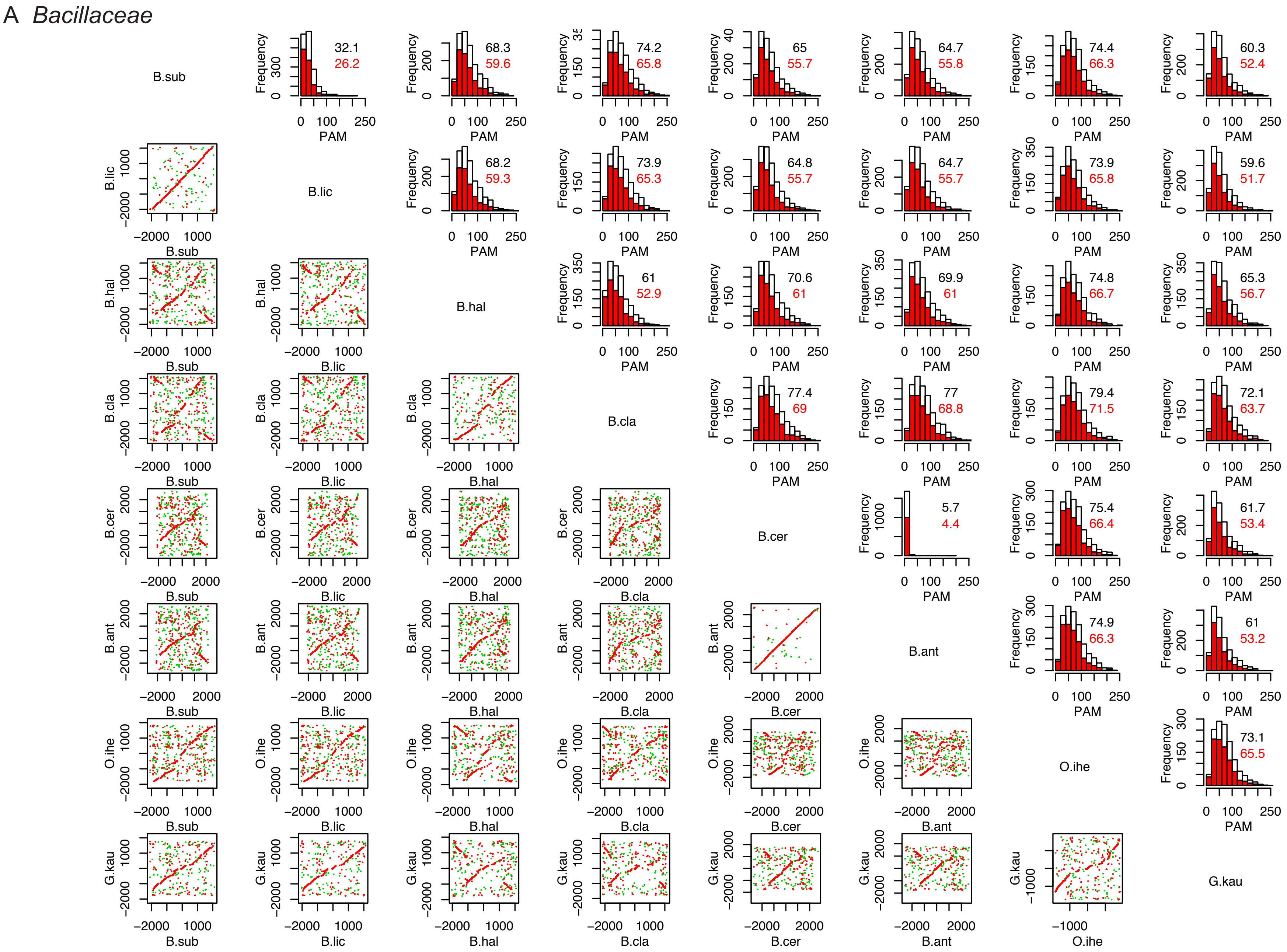

Figure S1. Pairwise comparisons between the organisms considered here in (A) *Bacillaceae* and (B) *Enterobacteriaceae*. The upper-right half shows histograms of the evolutionary distances (expressed in PAM units) between orthologs. The red bars show the histogram of the core genes. The black and red numbers in each histogram are the average PAM distances of all orthologs and the core orthologs, respectively, of each genome pair. The lower-left half shows dotplots for comparison of the positions of orthologous gene pairs. Core and non-core genes are indicated by red and green dots, respectively.

B *Enterobacteriaceae*

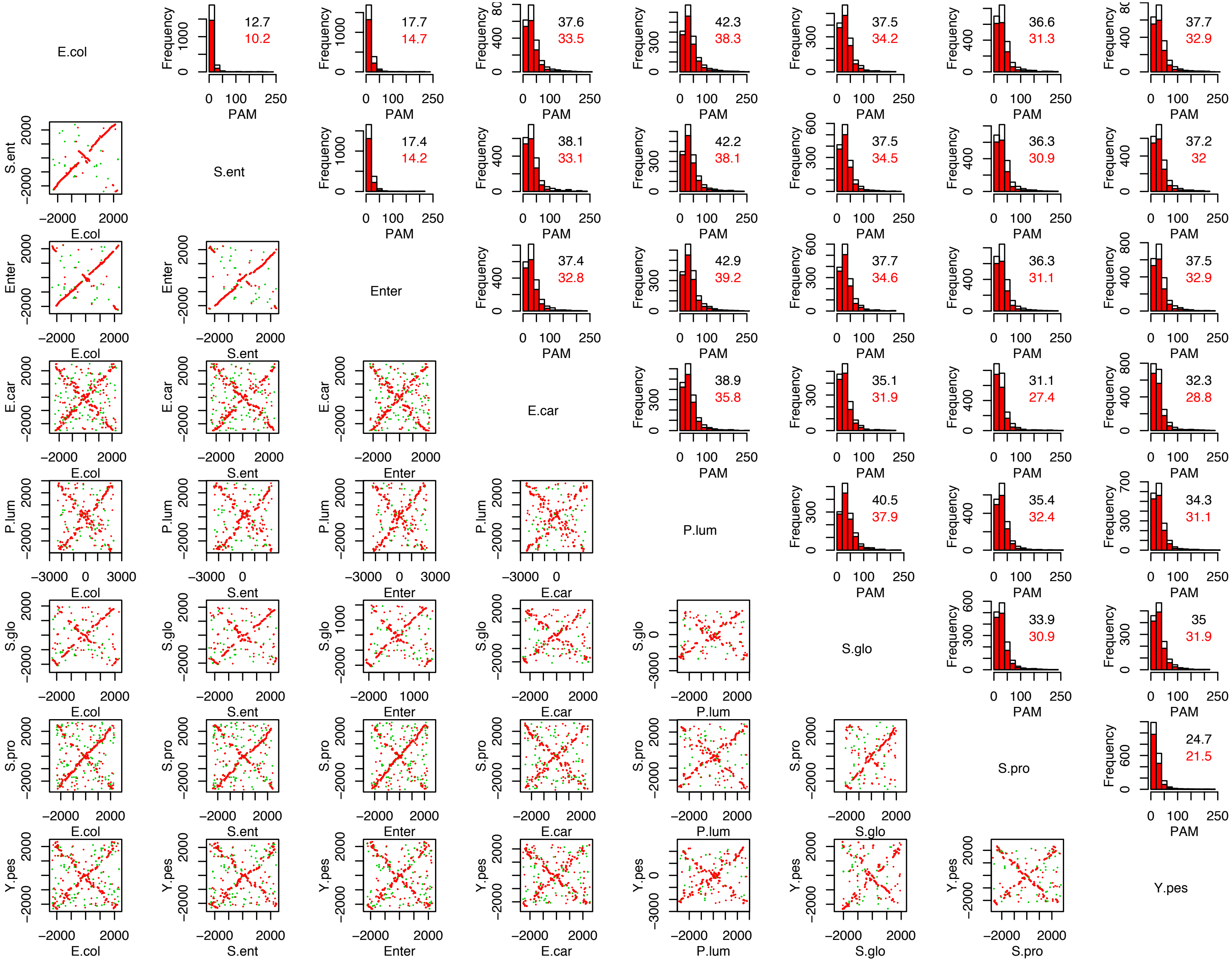

Figure S1. *(continued from the previous page)*
