# Supplementary figures and images for: Multiple genome alignment for identifying the core structure among moderately related microbial genomes
Source: BMC Genomics. 2008 Oct 31;9:515. doi: 10.1186/1471-2164-9-515 (PMC2615449; doi:10.1186/1471-2164-9-515)

## A *Bacillaceae*

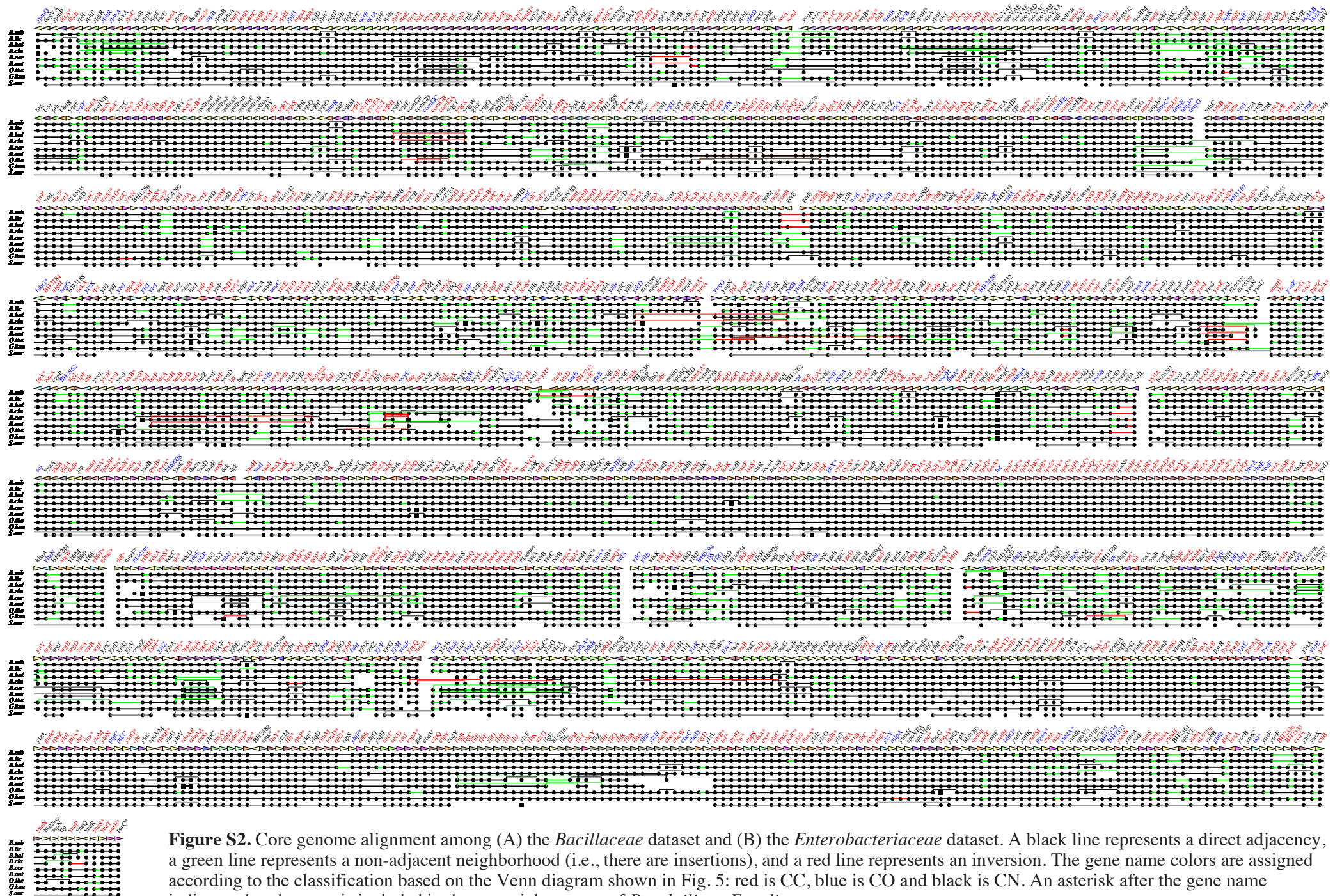

B *Enterobacteriaceae*

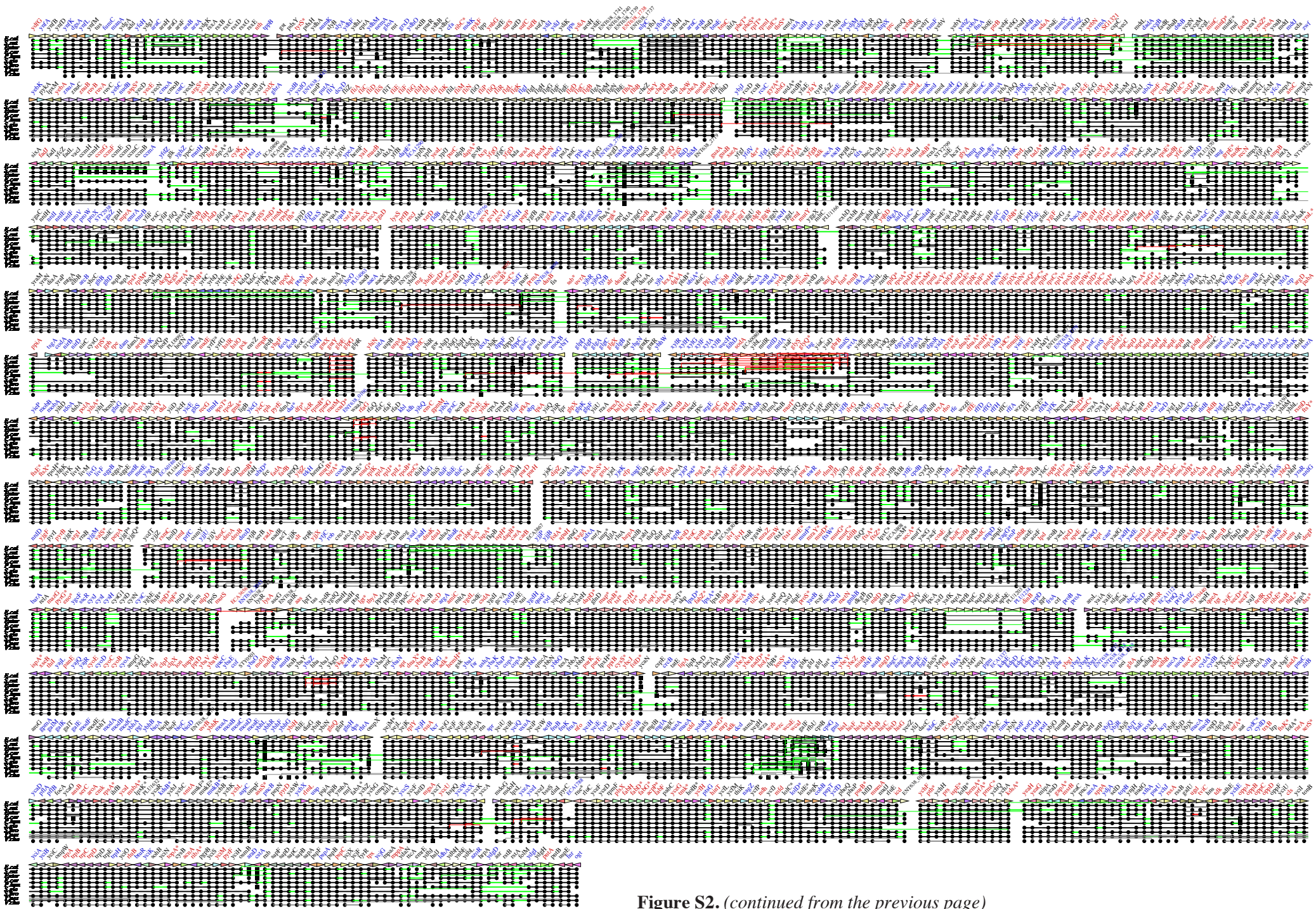

Figure S2. (continued from the previous page)

Supplement: Additional file 2 — Complete figures of the core genome alignments. [file 1471-2164-9-515-S2.pdf]
