# Supplementary material for: Multiple genome alignment for identifying the core structure among moderately related microbial genomes
Source: BMC Genomics. 2008 Oct 31;9:515. doi: 10.1186/1471-2164-9-515 (PMC2615449; doi:10.1186/1471-2164-9-515)
Supplement: Additional file 4 — Essential genes identified in the B. subtilis and E.coli genomes. [file 1471-2164-9-515-S4.pdf]

B. subtilis essentaial genes

	core-universal	core-conserved	noncore-universal	noncore-conserved	nonconserved	unique
Metabolism	eno fbaA pdhA pgm prs tkt gcaD glmS murB accA accB accC accD folD glyA yurV fabD fabF plsX ispE cdsA gpsA pgsA adk gmk guaB hprT cmk pyrG trxB yumC gatA gatB gatC gltX alaS alr asnS aspS asd serS fmt metK cysS ileS leuS valS dapA dapB dapF lysS murE ykuR argS hisS pheS pheT tyrS murC murD mraY murG csd yrvO yqeJ birA dfrA yqiD	pgk yvyH dxr dxs yacM yacN yqfP yqfY glyQ glyS murF proS trpS acpS menA menB menD menE	odhB nrdE nrdF nadE menC	ppaC tagD yueK		
Genetic Information Processing	rpoA rpoB rpoC sigA nusA rplA rplB rplC rplD rplE rplF rplI rplJ rplL rplM rplN rplO rplP rplQ rplR rplS rplT rplU rplV rplW rplX rpmA rpmB rpmC rpmD rpmF rpmH rpmI rpmJ rpsB rpsC rpsD rpsE rpsF rpsG rpsH rpsI rpsJ rpsK rpsL rpsM rpsN rpsO rpsP rpsQ rpsR rpsS rpsT rpsU cca cspR rnpA trmD trmU yqeI ywlC ffh ftsY secA secE secY prsA resB dnaE dnaN dnaX holB polC yqeN dnaA dnaB dnaC dnaG dnaI gyrA gyrB parC parE priA ssb topA dnaD hbs ligA pcrA	rpmGA map			rpmE	
Environmental Information Processing	mrpA mrpD yycF yycG	mrpB mrpC mrpF		tagG tagH		
Cellular Processes	ftsA ftsZ mreB mreC smc yacA ypuG	ftsW				
Others	groEL acpA ddl divIB divIC era fabG frr ftsL fusA gcp groES hepS hepT infA infB infC menH metS murAA obg pbpB pfkA prfA racE resA resC rnc spoVC tmk trxA tsf tufA ybbT ydiB ydiC yerQ yhdO yjbN ykqC ykuQ ylaN yloQ ylqF ymdA yneS yphC ypuH yqeH yqjK ysxC ytaG yurU yurX yurY	prfB rpmGB tpiA	rodA	tagA tagO ydiP yhdL ymaA	tagB tagF	ydiO

E. coli essentail genes

	core-universal	core-conserve	noncore-universal	noncore-conserved	nonconserved	unique
Metabolism	eno fbaA gapA pgk prsA glmS glmU murA murB accA accB accC accD folD suhB cydA* ppa can acpP fabA fabB fabD fabG fabZ dxr dxs ispD ispE ispF ispG ispH cdsA gpsA plsB plsC psd pssA adk gmk nrdA nrdB purB* spoT dut pyrG pyrH tmk glnS murI asnS aspS asd glyQ serS thrS fmt metG metK cysS leuS valS dapA dapB dapD dapE murE murF argS proS hisS trpS pheS pheT tyrS murC murD kdsA kdsB kdtA lpxA lpxB lpxC lpxD lpxH lpxK ftsI mraY murG lgt thiL ribA ribB ribC ribD ribE ribF nadD yfjB acpS coaD dfp birA folA folC folE folK hemA hemB hemC hemD hemG hemH hemL lolB prmC ubiA ubiB* ubiD* ispA ispU tadA*		pgsA gltX	fabI nadE entD*	rfaK*	
Genetic Information Processing	rpoA rpoB rpoC rpoE* rpoH lexA nusA nusG rplB rplC rplD rplE rplF rplJ rplL rplM rplN rplO rplP rplQ rplR rplS rplT rplU rplV rplW rplX rpmA rpmB rpmC rpmD rpmH rpsA rpsB rpsC rpsD rpsE rpsG rpsH rpsI rpsJ rpsK rpsL rpsM rpsN rpsP rpsQ rpsR rpsS cca map pth rnc* rne rnpA trmD yrdC ffh ftsY lepB lspA secA secD secE secF secY yidC dnaE dnaN dnaX holA holB dnaA dnaB gyrA gyrB parE ssb topA ligA yqgF			dnaC		tdcF*
Environmental Information Processing	cydC* ftsE ftsX lolC lolD lolE msbA mviN ftsK csrA			tnaB*		
Cellular Processes	ftsA ftsB* ftsH ftsL ftsN ftsQ ftsW ftsZ minD* minE* mrdB mreB mreC mreD mukB mukE mukF tilS zipA					
Others	def degS der era fldA frr fusA groS grpE imp infA infB ispB lnt lolA mrdA obgE orn prfA rlpB secM tsf wzyE* yadR yaeL yaeT yeaZ yejM yfiO* ygjD yhbN yhhQ* yigP* yihA yjeE yjgP yjgQ yraL* yrbB* yrbK yrfF	infC yagG* yhbV*		bcsB* chpR* chpS* dicA* ygiT* ymfK*	yceQ* yefM*	alsK* racR* ydfB* ydiL* yqgD*

Table S2. Essential genes identified in the *B. subtilis* and *E. coli* genomes. The functional classification is based on the COG database. The essential genes included in the report by Baba et al. (2006) but not in that by Kato and Hashimoto (2007) are indicated with an asterisk.
