# Supplementary material for: Multiple genome alignment for identifying the core structure among moderately related microbial genomes
Source: BMC Genomics. 2008 Oct 31;9:515. doi: 10.1186/1471-2164-9-515 (PMC2615449; doi:10.1186/1471-2164-9-515)
Supplement: Additional file 5 — Global metabolic map displaying the common core genes shared between Bacillaceae and Enterobacteriaceae drawn by the KEGG Atlas system. [file 1471-2164-9-515-S5.pdf]

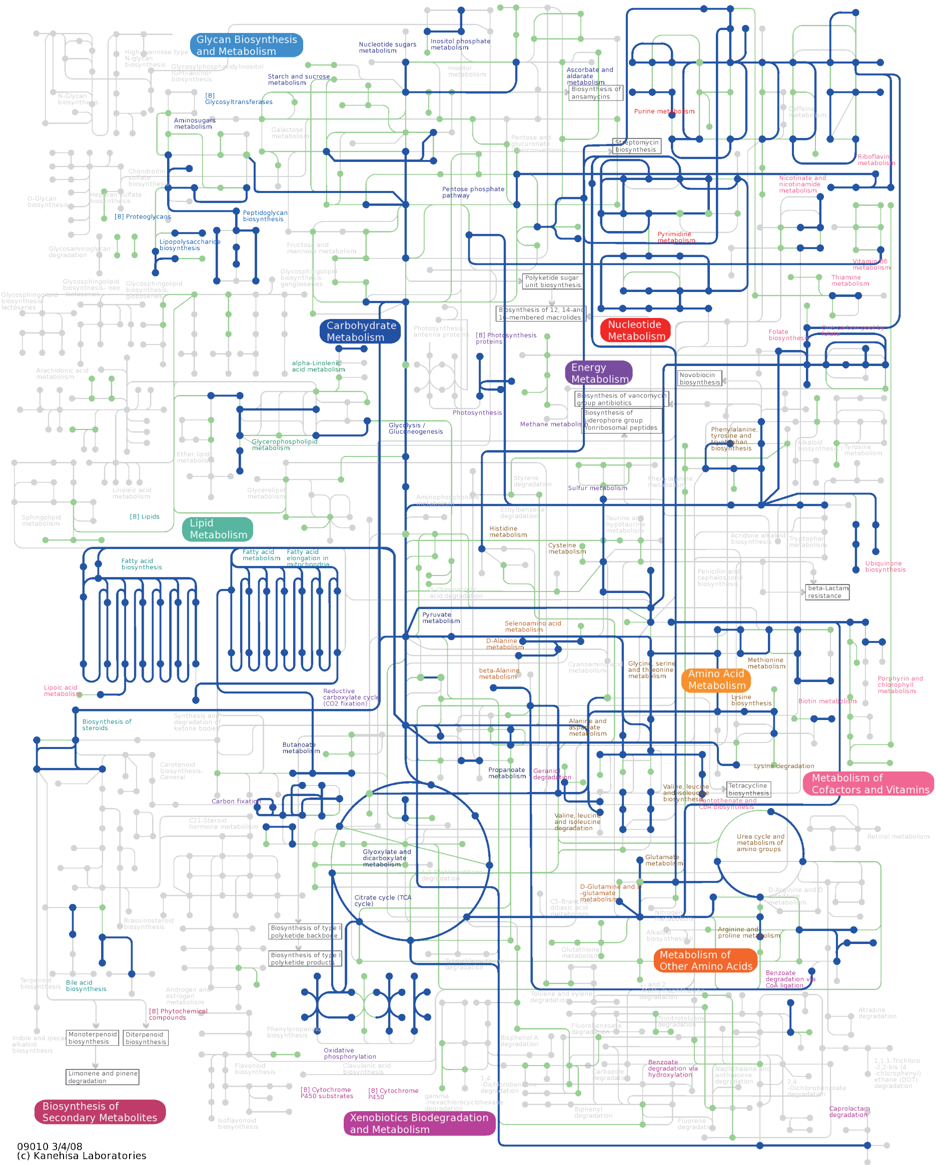

Figure S3. Global metabolic map displaying the common core genes shared between *Bacillaceae* and *Enterobacteriaceae* (CC class), which was drawn by the KEGG Atlas system (Kanehisa et al., 2008). The map was drawn based on the *E. coli* genes. The blue dots and lines indicate the pathway generated from the *E. coli* genes belonging to the common core OGs, and the green dots and lines indicate the entire *E. coli* pathway.
