# Supplementary material for: Multiple genome alignment for identifying the core structure among moderately related microbial genomes
Source: BMC Genomics. 2008 Oct 31;9:515. doi: 10.1186/1471-2164-9-515 (PMC2615449; doi:10.1186/1471-2164-9-515)
Supplement: Additional file 6 — Means and standard deviations of the GC3(%) values in each class. [file 1471-2164-9-515-S6.pdf]

Table S3. Means and standard deviations of the GC3 (%) values in each class.

	A	B	C	D	E	F
B. anthracis	26.49±3.78	26.83±3.98	26.35±4.16	26.05±4.09	26.16±4.39	25.58±5.12
B. cereus	26.18±3.8	26.4±4.07	25.92±4.08	25.9±4.29	25.92±4.41	25.49±5.02
B. clausii	44.7±3.71	43.9±3.87	44.23±4.63	44.09±5.5	42.83±6.9	42.06±7.76
B. halodurans	43.02±4.47	42.49±4.19	43.64±4.92	42.78±5.16	41.97±5.93	39.93±6.56
B. licheniformis	52.78±4.66	52.72±4.62	53.66±5.47	51.83±6.6	50.09±7.37	45.68±9.9
B. subtilis	44.5±4.93	44.36±5.22	47.31±5.81	45.63±7.04	43.78±8.26	38.01±9.81
G. kaustophilus	65.55±4.89	65±5.82	62.29±8.79	60.21±10.68	58.54±11.83	55.29±12.61
O. iheyensis	24.46±3.52	24.45±3.51	26.14±3.9	26.35±4.47	26.69±4.57	26.99±5.37
E. coli	56.93±5.32	56.8±5.9	54.2±9.39	55.5±8.08	52.99±9.94	49.41±11.24
S. enterica	60.89±4.96	60.86±7.19	58.83±7.41	58.59±8.47	55.81±10.05	48.22±12.05
Enterobacter sp.	60.03±5.6	61.98±6.09	62.32±7.29	62.22±7.97	59.78±9.36	53.44±12.47
E. carotovora	56.33±5.57	57±6.71	57.76±7.26	57.88±7.35	54.54±10.68	51.24±12.19
P. luminescens	39.45±4.81	40.03±5.98	37.86±8.7	40.66±9.92	40.17±11.65	37.04±10.41
S. glossinidius	67.87±5.43	65.54±6.22	65.52±7.86	59.88±10.72	58.76±8.91	58.12±10.29
S. proteamaculans	66.68±5.25	67.59±6.34	67.45±7.16	66.86±7.59	64.9±9.28	62.19±11.4
Y. pestis	49.14±5.39	49.8±5.62	50.74±7.68	50.97±7.72	49.6±9.59	44.04±10.32
Average[1]	47.58±4.69	47.66±5.21	47.58±6.43	47.09±7.04	45.72±8.07	43.07±9.13

[1] The simple arithmetic average of the means and the standard deviations of the 16 organisms examined rather than the mean and the standard deviation of the combined dataset of these organisms.
