# Supplementary material for: Multiple genome alignment for identifying the core structure among moderately related microbial genomes
Source: BMC Genomics. 2008 Oct 31;9:515. doi: 10.1186/1471-2164-9-515 (PMC2615449; doi:10.1186/1471-2164-9-515)
Supplement: Additional file 7 — Proportions of genes with significantly deviated GC3 values in each class. [file 1471-2164-9-515-S7.pdf]

Table S4. Proportions of genes with significantly deviated GC3 values in each class.

	Core GC3 (%)		Significantly deviated genes / All genes (%)					
	mean	sd	A	B	C	D	E	F
B. anthracis	26.56	3.82	0.64	0.90	1.1	0.362	0.626	1.65
B. cereus	26.23	3.86	0.258	0.858	0.804	0.868	0.794	1.94
B. clausii	44.51	3.76	0.238	1.57	2.27	4.39	10.3	13.8
B. halodurans	42.89	4.41	0.714	0.687	0.709	1.59	3.05	6.54
B. licheniformis	52.76	4.65	0.471	0.327	1.88	4.15	7.38	20.9
B. subtilis	44.46	5.01	0.121	0.326	0.645	2.06	6.19	19.3
G. kaustophilus	65.41	5.15	0.517	1.11	10.5	13.8	18.2	24.7
O. iheyensis	24.45	3.52	0.719	0.922	1.08	3.04	4.18	6.07
E. coli	56.88	5.56	0.291	0.718	7.69	3.97	8.88	19.2
S. enterica	60.87	5.95	0	2.63	0	5.09	9.98	31.1
Enterobacter sp.	60.82	5.88	0.0951	0.422	0.855	2.16	4.59	18.3
E. carotovora	56.60	6.07	0.371	0.683	0	1.50	8.15	15.9
P. luminescens	39.63	5.21	0.0923	0.612	5.36	7.57	9.18	9.97
S. glossinidius	67.51	5.62	0.527	0.966	5.56	16.8	14.7	19.2
S. proteamaculans	67.07	5.75	0.274	1.25	2.51	3.1	6.95	12.0
Y. pestis	49.40	5.49	0.183	0.587	1.52	3.54	6.88	11.9
Average[1]	47.65	4.90	0.371	0.925	2.71	4.43	7.26	13.39

[1] The simple arithmetic averages of the values of the 16 organisms examined rather than the values calculated against the combined dataset of these organisms.
