# Supplementary material for: Multiple genome alignment for identifying the core structure among moderately related microbial genomes
Source: BMC Genomics. 2008 Oct 31;9:515. doi: 10.1186/1471-2164-9-515 (PMC2615449; doi:10.1186/1471-2164-9-515)
Supplement: Additional file 8 — A problematical case found during an experimental core structure extraction from the Enterobacteriaceae dataset. [file 1471-2164-9-515-S8.pdf]

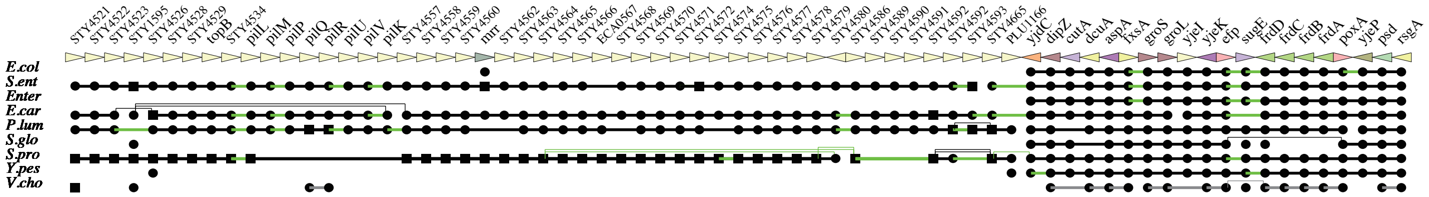

Figure S4. A problematical case found during an experimental core structure extraction from the *Enterobacteriaceae* dataset. The left-side region next to the *yjdC* gene is probably a part of a genomic island, and was finally removed by the procedure that eliminates locally non-conserved regions in step 5 of the CoreAligner procedure.
